# The effects of supported housing for individuals with mental disorders

**DOI:** 10.1002/hec.4579

**Published:** 2022-08-19

**Authors:** Francisca Vargas Lopes, Pieter Bakx, Sam Harper, Bastian Ravesteijn, Tom Van Ourti

**Affiliations:** ^1^ Department of Public Health Erasmus MC Rotterdam The Netherlands; ^2^ Erasmus Centre for Health Economics Rotterdam Erasmus University Rotterdam Rotterdam The Netherlands; ^3^ Erasmus School of Health Policy and Management Rotterdam The Netherlands; ^4^ Department of Epidemiology, Biostatistics & Occupational Health McGill University Montreal Quebec Canada; ^5^ Erasmus School of Economics Erasmus University Rotterdam Rotterdam The Netherlands; ^6^ Tinbergen Institute Rotterdam The Netherlands

**Keywords:** instrumental variable, leniency design, long‐term care, mental health, policy evaluation, supported housing

## Abstract

Societies face the challenge of providing appropriate arrangements for individuals who need living support due to their mental disorders. We estimate the effects of eligibility to the Dutch supported housing program (*Beschermd Wonen*), which offers a structured living environment in the community as an intermediate alternative to independent housing and inpatient care. For this, we use exogenous variation in eligibility based on conditionally random assignment of applications to assessors, and the universe of applications to supported housing in the Netherlands, linked to rich administrative data. Supported housing eligibility increases the probability of moving into supported housing and decreases the use of home care, resulting in higher total care expenditures. This increase is primarily due to the costs of supported housing, but potentially also higher consumption of curative mental health care. Supported housing eligibility reduces the total personal income and income from work. Findings do also suggest lower participation in the labor market by the individuals granted eligibility, but the labor participation of their parents increases in the long‐run. Our study highlights the trade‐offs of access to supported housing for those at the margin of eligibility, informing the design of long‐term mental health care systems around the world.

## 1 INTRODUCTION

Individuals with mental disorders often lead unstable lives in terms of their social and physical living environment. Severe mental illness is associated with economic inactivity (Ettner et al., 1997; Hewlett & Moran, 2014; Sturm et al., 1999), social insurance benefit receipt (Kouzis & Eaton, 2000), having limited family relationships (Place & Michon, 2014) and homelessness (Fazel et al., 2008; Sullivan et al., 2000), and family members might experience negative health and labor market consequences (Carmichael & Charles, 2003). Inpatient mental health treatment offers curative or long‐term clinical care, which is costly and limits autonomy (Chilvers et al., 2006; Fakhoury & Priebe, 2007). On the other hand, living independently means limited structure and support, which may contribute to mental health deterioration. Supported housing offers an alternative, intermediate model, typically consisting of a structured, semi‐independent living environment without mental health treatment, which may be used on an outpatient basis. However, there is currently limited evidence on the effect of such living arrangements on health care use and other outcomes (Chilvers et al., 2006; McPherson et al., 2018a).

Supported housing in the Netherlands offers a comprehensive package, including housing, personal and domestic care, guidance, and daily activities. Mental health treatment is not provided by supported housing but can be obtained in the form of regular outpatient treatment. Supported housing is meant for a broad range of individuals who need a well‐structured living environment (CIZ, 2014), including those with personality disorders such as borderline, autism spectrum disorder, individuals who have recovered from substance use disorder, schizophrenia, attention‐deficit/hyperactivity disorder, mood disorder, and behavioral problems with aggression (BeschermdWonen.nl., 2019). This group includes homeless individuals but also individuals who lived in an inpatient institution or in a private household before. Supported housing and other types of care are accessible regardless of income: the Netherlands has generous coverage of treatment and support for individuals with mental disorders. During the study period, outpatient and inpatient mental health treatment up to a year were covered as universal health benefits through mandatory private health insurance, while home care, supported housing and long‐term inpatient mental health treatment were universally covered through public long‐term care insurance with low levels of patient cost‐sharing (Bakx et al., 2020).

In this paper, we estimate the effects of eligibility for the publicly funded Dutch supported housing program (*Beschermd Wonen*) on care use, mortality, employment and income of the people with mental disorders, and on spillovers to their parents. In the study period, eligibility was determined by an independent national agency. We use data on nearly 8,000 applications to supported housing by adults younger than 80 years old, between 2011 and 2013. The data include the outcome of the assessment and links all applications to their assessor. These application data were linked to administrative data on demographic characteristics, long‐term care use, mental health treatment records and mortality of the applicants; and to health insurance claims and economic outcomes of both applicants and their parents. We use conditionally random assignment of applications to the agency's assessors who determine eligibility for subsidized supported housing, and, importantly, who vary in terms of their leniency (Bakx et al., 2020; Dahl et al., 2014; Maestas et al., 2013). An application who was assigned to a strict assessor may be determined ineligible for supported housing, while the same application would have been eligible if assigned to a lenient assessor. We estimate local average treatment effects (LATE) of eligibility for supported housing among individuals whose eligibility was affected by the random variation in assessor leniency using an instrumental variables design.

Our estimates suggest that, among individuals whose eligibility depends on assessor assignment, supported housing eligibility increases the probability of moving into supported housing by 31.6% points (pp) (standard error (se): 5.7) in the next calendar year, and decreases the likelihood of using home care by 13.4 pp (se: 6.4). Average total care expenditure increases by 20,017 euros (se: 5,006), mostly at the expense of supported housing expenditure (11,883 euros; se: 2,634), and potentially also mental health treatment costs (7,698 euros; se: 6,440). We find little evidence of substantial offsetting cost savings on other types of care. We also find that eligibility for supported housing decreases personal income by 1,470 euros (se: 748) in the short‐run and 2,017 euros (se: 747) in the long‐run, as well as income from work by 2.149 euros (se: 717) and 3,410 euros (se: 1,488), and likely extensive‐margin labor participation by 7.0 pp (se: 3.6) and 7.5 pp (se: 5.5.), respectively. On the other hand, both mothers and fathers of applicants increase their labor participation in the fourth year after their child is granted eligibility to supported housing, by 15.1 pp (se: 7.2) and 17.8 pp (se: 7.6), respectively.

Our results are consistent with previous findings in the literature which suggest that a stable living environment supports mental health treatment adherence (Gilmer et al., 2010, 2014). We do not find evidence that eligibility for supported housing decreases mental health treatment costs overall, as suggested by previous studies which argued that supported housing may reduce costly service use, such as acute hospitalizations, by preventing severe mental health deterioration (Malinovsky et al., 2013; McDermott et al., 2016; Muir et al., 2008). The positive effects of supported housing eligibility on parental extensive‐margin labor supply may reflect that supported housing is a substitute for informal care by family members. Disability has been found to negatively impact parents' labor market outcomes, both in terms of participation and earnings (Cidav et al., 2012; Eriksen et al., 2021), with smaller effects on fathers compared with mothers (Gunnsteinsson & Steingrimsdottir, 2019; Kvist et al., 2013; McCall & Starr, 2018).

Our study adds to the existing evidence by reporting causal effects for the broader population with mental health problems, and not focusing on specific groups such as the homeless living with mental disorders or patients undergoing deinstitutionalization after long‐periods living in institutions (McPherson et al., 2018a). Another key strength of our design is that our estimates apply to individuals for whom supported housing eligibility is determined by the leniency of the assessors. This provides evidence relevant for policy‐makers because determination of eligibility for supported housing, rather than the individual decision to apply, is the primary policy lever for affecting uptake of supported housing in the Netherlands. The individuals at the margin of eligibility in our study correspond to the group most likely impacted by policy changes entailing a shift in the eligibility threshold for supported housing.

This paper proceeds as follows: Section 2 provides information on supported housing in the Netherlands and the application procedure. Section 3 describes the rich administrative data sources which were linked at the individual level for the purpose of this study. Section 4 describes the use of assessor leniency as a source of exogenous variation in eligibility for supported housing. Section 5 describes the results and Section 6 discusses these results and concludes.

## 2 INSTITUTIONAL BACKGROUND

### 2.1 Care for people with mental disorders in the Netherlands

During the study period, public long‐term care insurance was funded through the Exceptional Medical Expenses Act and covered three types of care for people with mental disorders or psychosocial problems: home care, supported housing^1^ and institutional care. Home care could be nursing, personal care (e.g., hygiene), or individual or group guidance.^2^ Institutional care consisted of long‐term inpatient stays beyond 365 days, and was the only setting in which clinical treatment was funded by the public long‐term care insurance. For the remaining individuals –even those receiving publicly funded home care or supported housing– mental health treatment was funded through mandatory private health insurance and provided by mental health care providers in an outpatient setting or through shorter inpatient stays (up to 365 days). General practitioners acted as gatekeepers for mental health services and provided basic support to mild complaints of mental health distress.

### 2.2 Supported housing

Supported housing is typically provided to individuals after inpatient mental health care, deterioration of mental health while living independently in the community, or as a way to move out of community‐based rehabilitation units or forensic services. It aims at addressing functional impairment, developing living skills, and improving social functioning. These principles may be implemented in various forms and supported housing providers might differ considerably in terms of physical structure, staffing arrangements, and degree of support (McPherson et al., 2018a).

Supported housing aims at providing a stable environment, daily regularity and meaningful daily activities, such as different types of working experiences or non‐work‐related activities to provide rhythm and regularity (e.g., taking a group walk, doing crafts or helping with farm activities in a rural environment). The level of support provided varies in intensity and depends on the individual needs, including medication management, personal care, household chores such as cooking and cleaning, and financial administration. Besides the practical assistance there is also a focus on rehabilitating individuals to manage these tasks and achieving personal goals in social and occupational domains. Individuals might be incentivized to different levels of participation in the labor market, ranging from voluntary work to sheltered or competitive employment. As mentioned in the previous section, supported housing in the Netherlands does not include any clinical care, which individuals can receive in the outpatient setting from mental health care providers. The fact that supported housing intends to proxy living in the community (CIZ, 2014) and is fully separated from mental health services is a key aspect in distinguishing it from institutional care such as a hospitalization or living in a closed ward (Fakhoury & Priebe, 2007).

During our study period most supported housing in the Netherlands would be offered by 21 large providers dedicated to this type of housing services [Dutch Regional Institutes for Supported Housing–Regionale Instelling voor Beschermd Wonen (RIBW)] or by providers from the mental health care system that would have clinical care as their core activity (De Heer‐Wunderink et al., 2012). The accommodations would be typically organized as grouped apartments, with or without a shared living room and kitchen, or as one‐family homes that provide single bedrooms and a shared bathroom, kitchen and living room (usually for four residents). Staff would be available on‐site up to 24 hours a day, and individuals would be allocated to a care coordinator, skilled in psychiatric rehabilitation support (De Heer‐Wunderink et al., 2012; Roeg et al., 2021). According to the Simple Taxonomy for Supported housing (STAX‐SA) (McPherson et al., 2018b) most of these accommodation‐based services would reflect type 2 and type 3 services: congregate setting, high to moderate support, strong emphasis on move‐on (Roeg et al., 2021). Around the same period, there was also an increase in other type of organizations offering supported housing, namely small‐scale housing facilities and care farms, pioneered in the Netherlands (De Heer‐Wunderink et al., 2012; Hassink et al., 2010).

Individuals admitted paid a monthly co‐payment adjusted to their age, income and family situation, and to the number of days in supported housing (but not on the intensity of the support). Co‐payments ranged from 0 to 2,300 euros per month, but never exceeded income. People who received home care paid a lower copayment that depended on the same parameters but also on the amount of care used.

### 2.3 Determination of supported housing eligibility

Throughout the study period, individuals seeking long‐term care in the Netherlands had to apply for eligibility at an independent needs‐assessment agency (*Centrum Indicatiestelling Zorg* ‐ CIZ). Applications were submitted to a regional office of the agency by the individual or by proxy, such as a family member, social worker or care provider. The application contained information about health problems, functional limitations, background characteristics, and the type of care applied for.

Applications were assigned to assessors by planners within each regional office, based on the assessors' contemporaneous workload. Information about health or care needs was not taken in consideration by the planner for allocating applications. The traveling time of assessors and foreign language fluency could be taken into account to determine assessor assignment. Assessors had considerable discretionary power to apply generic rules to specific cases: within pre‐set boundaries, they decided which additional information to collect and how to reach a decision. The assessor decided about (a) the type of long‐term care, (b) the intensity of care and (c) the duration of eligibility, ranging between several months and 15 years. When the eligibility period finished there should be a new application; individuals could also submit a new application at any time.

The application was first screened to determine its validity and, if so, whether it could be approved by a back office employee^3^ or whether it should be reviewed by an assessor. In the latter case, the screener also determined the type of assessment procedure: abridged or extended.^4^ An abridged assessment procedure consisted of desk research and phone interviews. Information used by the assessor included the information filled out on the application form and, if applicable, information about prior long‐term care use or information collected in previous applications. The assessor could additionally collect or verify information via phone interview with the individual, household and family members, the health insurer, and care providers. On top of this, an extended assessment procedure would include a face‐to‐face interview and a review by a multidisciplinary team, including medical professionals. Most applications would be defined as regular and handled within 6 weeks, with a smaller number of applications classified as priority and being handled in a shorter time‐frame.

Importantly, the scope of the needs‐assessment agency would be limited to the eligibility decision, with assessors having no further interaction with applicants or providers about the actual admission to supported housing. Providers of supported housing are private non‐for‐profit organizations, often specialized in providing services to particular diagnoses (e.g., autism, personality disorders or substance use problems), psychosocial problems or dual diagnosis (e.g., mild intellectual disability in combination with a mental disorder). Providers could specify entry criteria beyond eligibility (to ensure a good match between their specialization and the needs of their clients) and set limits to the duration of services provision.

## 3 DATA

### 3.1 Data sources

We use pseudonymized administrative data from CIZ on all applications to long‐term care in the Netherlands between 2009 and 2013. The CIZ data include information about the application (date, person filing the application, type of application, type of assessment procedure and type of care and intensity requested), eligibility decision (type of care, intensity, and duration attributed) and a pseudonymized assessor identifier. Application data is linked to nationwide administrative records at the individual level. Linked datasets include (a) municipal registries (2009–2017) for demographics, household composition and linkage to parents; (b) data on long‐term care use (2010–2014) for information on admissions to supported housing or other long‐term care institutions and use of home care; (c) specialist mental health treatment records (2010–2014) for information on mental disorders diagnosis, available for applicants treated by mental health care specialists; (d) health insurance claims (2010–2014) for data on annual health care expenditures of the applicants and the parents (including curative outpatient and inpatient mental health care expenditure); (e) tax records (2010–2017) for income from work and total personal income (gross labor earnings plus transfers) of the applicants and the parents; and, (f) death records (2011–2017) for all‐cause mortality of the applicants.

### 3.2 Study population

Starting from all long‐term care applications in the Netherlands between 2009 and 2013 we construct a homogeneous and relevant population to measure the leniency of the assessors. First, we select applications who requested supported housing. Second, we exclude applications of individuals older than 79 and people who identified as having a psychogeriatric illness or a disability, or who requested palliative care. Third, we exclude applications in which the assessors’ discretionary power would be absent or limited: applications approved by back‐office employees, emergency applications handled in shorter time‐frames, applications that follow specific predefined routes, and applications of individuals that obtained a valid supported housing eligibility decision during the previous 365 days.^5^ Finally, to obtain precise estimates of assessor leniency, we restrict our data to applications that were handled by assessors with at least 30 applications of the same regional office and assessment procedure within the study period. Table S1 in the appendix shows that most exclusion criteria impacted fewer than 6% of the applications. The exceptions are applications of individuals eligible to supported housing in the last 365 days (27.0%) and applications of assessors below the minimum threshold of 30 applications (17.8%). The impact of the latter two exclusions in our results was assessed in robustness analyses.

After exclusions we obtain 12,767 applications done between 2009 and 2013, who were assigned to 198 assessors. These applications are used to construct the leniency instrument. The instrumental variable estimation is performed on a smaller population of 7,953 applications done between 2011 and 2013 and corresponding to 187 assessors (from here onwards referred to as the study population). Applications from 2009 to 2010 are not included in the estimation due to limitations in the administrative data available for several outcomes and control variables of interest.^6^

### 3.3 Descriptive statistics

Most applications in our study population are from men (71%) and about 28% comes from first‐ or second‐generation migrants (Table 1). Mean age at the time of the application was 38 years and most individuals lived alone (46%), in an institution (20%), or with their parents (both parents 15%, single parent 8%). Twenty‐five percent had a paid job in the prior calendar year, and the average total personal income was 12,413 euros, which is in the third income decile for the entire population (CBS, 2021). The most common mental disorder diagnoses are substance use disorders (24%), psychotic disorders (19%) or disorders diagnosed during childhood (13%).^7^ Applications were most often submitted by long‐term care providers (79%) and judged through an extended assessment procedure (76%). 34% of the applications were already eligible for another type of long‐term care in the month prior to application. The vast majority of applications (95%) were made by individuals who applied to supported housing only once during the study period, and 86% were granted eligibility for supported housing. About 8% of the applications were granted eligibility for home care, which mostly consisted of individual guidance and/or group guidance; 6% were not granted eligibility for any (long‐term) care; and fewer than 1% were assessed as eligible to institutional care (elderly nursing homes, institution for the disabled or inpatient mental health facilities).

**TABLE 1:** Descriptive statistics: group means for study population and by supported housing eligibility

	Total mean (sd)	Eligible mean (sd)	Non‐eligible mean (sd)	Difference: Non‐eligible versus eligible	*p*‐Value *t*‐test
**Individual**					
Female	0.29	0.30	0.29	−0.01	0.668
Age	37.73 (15.42)	37.92 (15.54)	36.57 (14.65)	−1.34	0.007
Dutch background	0.73	0.73	0.71	−0.02	0.106
Western migration background^a^	0.10	0.10	0.09	−0.01	0.350
Non‐western migration background^a^	0.18	0.17	0.20	0.03	0.009
Living alone	0.46	0.46	0.44	−0.02	0.187
Living with partner	0.07	0.07	0.09	0.03	0.001
Living with parents	0.15	0.15	0.13	−0.02	0.057
Living with single parent	0.08	0.08	0.08	0.00	0.754
Living institution	0.20	0.20	0.18	−0.02	0.135
Other position in household	0.05	0.04	0.07	0.03	0.000
Prior^b^ personal income (€)	12,413 (7,555)	12,288 (7,403)	13,164 (8,378)	876	0.000
Prior^b^ working	0.25	0.25	0.31	0.06	0.000
Prior^b^ specialist mental health care	0.67	0.68	0.59	−0.09	0.000
Prior^b^ home care	0.15	0.16	0.11	−0.05	0.000
Prior^b^ health care expenditure (€)	21,059 (39,932)	22,473 (40,590)	12,565 (23,356)	−9,908	0.000
Mental health disorder diagnosis^c^					
Substance use disorder	0.24	0.23	0.27	0.05	0.001
Psychotic disorder	0.19	0.21	0.08	−0.13	0.000
Disorder diagnosed in the childhood	0.13	0.14	0.10	−0.04	0.000
Mood disorder	0.08	0.08	0.08	0.00	0.873
Personality disorder	0.08	0.08	0.08	−0.00	0.682
Other diagnosis	0.10	0.09	0.11	0.04	0.000
No treatment/diagnosis	0.18	0.16	0.27	0.09	0.000
**Application**					
Application by long‐term care provider	0.79	0.80	0.73	−0.07	0.000
Application by social worker	0.14	0.13	0.19	0.06	0.000
Application by other^d^	0.07	0.07	0.08	0.01	0.318
Regular application	0.96	0.96	0.96	−0.00	0.896
Abridged assessment procedure	0.24	0.18	0.60	0.41	0.000
Extended assessment procedure	0.76	0.82	0.40	−0.41	0.000
Eligible for long‐term care last month^e^	0.34	0.34	0.32	−0.02	0.166
More than 1 application/individual	0.04	0.02	0.22	0.20	0.000
**Eligibility**					
Supported housing	0.86	1.00	0.00	−1.00	.
Inpatient mental health care	0.00	0.00	0.01	0.01	0.000
Elderly nursing home	0.00	0.00	0.03	0.03	0.000
Institution for the disabled	0.00	0.00	0.02	0.02	0.000
Negative decision	0.06	0.00	0.41	0.41	0.000
Any home care^f^	0.08	0.01	0.53	0.52	0.000
Nursing	0.01	0.00	0.03	0.03	0.000
Individual guidance	0.08	0.01	0.52	0.51	0.000
Group guidance	0.02	0.00	0.10	0.09	0.000
**Observations**	7,953	6,818	1,135		

Abbreviation: sd, standard deviation.

^a^ First‐generation migrants are classified according to their country of birth. Second‐generation migrants are classified according to their mother's country of birth–if that is the Netherlands the father's country of birth is considered.

^b^ Prior refers to the calendar year before the year of application.

^c^ Only available for those who receive specialist mental health care in the past 365 days.

^d^ Including herself.

^e^ Except for supported housing, as all those eligible for supported housing in the 365 days ahead of the application were excluded.

^f^ The rows below refer to the different types of home care granted in our population. Individuals can be eligible for more than one type of home care simultaneously.

Individuals who were eligible for supported housing differ from those considered as non‐eligible (Table 1). Most importantly, they are older, have a lower prior personal income, are less likely to have worked, and more likely to have used specialist mental health care or home care before, or to have a diagnosis of psychotic disorder or of a disorder diagnosed in the childhood. Eligible individuals also have much higher health care expenditures in the prior year. Some of the application characteristics also differ by eligibility: applications granted eligibility were more often done by long‐term care providers and were more often assessed through an extended procedure.

## 4 EMPIRICAL APPROACH

We estimate the effects of supported housing eligibility on a range of outcomes including long‐term and health care use, mortality, employment and income, and parental outcomes. This relationship is given by

(1) $$y_{i}=\alpha_{0}+\alpha_{1}Eligibility_{i}+Controls_{i}^{\prime }\alpha^{C}+v_{i}$$

Where $y_{i}$ is the outcome of interest for each application to supported housing *i*, $Eligibility_{i}$ corresponds to a binary indicator that takes the value 1 when the assessor decides that the applicant is eligible to receive supported housing, $Controls_{i}$ refers to a set of covariates related to the applicant and to the application, and $v_{i}$ is an error term. $\hat{\alpha_{1}}$ is likely biased because assessors grant eligibility based on characteristics that are partly unobserved to the researchers (e.g., unobserved dimensions of health, wellbeing, behavior, social context).

To mitigate bias in $\hat{\alpha_{1}}$ we use an instrumental variable approach that exploits exogenous variation in eligibility for supported housing arising from differences between assessors' leniency (Bakx et al., 2020; Dahl et al., 2014; Maestas et al., 2013). That is, we exploit the fact that (a) the application's eligibility status for supported housing was determined by an assessor of the independent needs‐assessment agency; (b) the allocation of applications to assessors was non‐systematic within the regional office and each type of assessment procedure; and (c) assessors had differential discretionary power, with some being systematically more likely to judge similar applications as eligible than others.

### 4.1 Leniency instrument

We estimate the assessors' leniency by calculating the share of other applications that they approved for the period of 2009 to 2013. This leave‐one‐out measure of leniency for application *i* handled by each assessor within a regional office *j* and using the type of assessment procedure *a* is calculated as the proportion of applications for which eligibility was granted

(2) $$Leniency_{ija}=\frac{Number\;of\;approvals_{ja}-I_{i}\left(approved\right)}{number\;of\;applications_{ja}-1}$$

**FIGURE 1:**
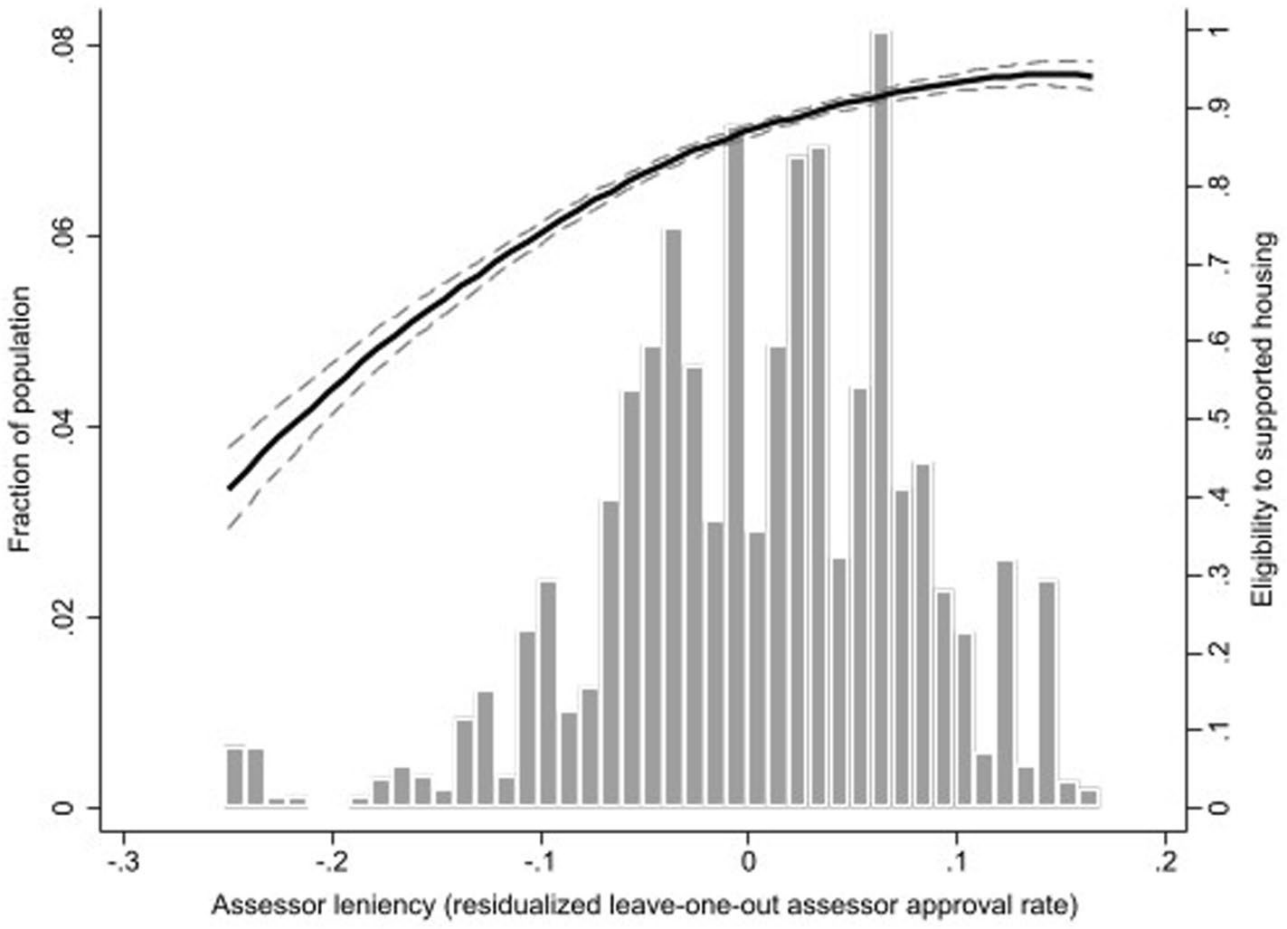
Distribution of assessors’ residualized leniency and its effect on eligibility to supported housing (first‐stage). Estimates of eligibility are obtained using a local linear regression of residualized leniency on each eligibility decision

Where each approved application $I_{i}\left(approved\right)$ is excluded to guarantee that the instrument is exogenous. We further calculate a residualized measure of leniency to ensure that our instrument does not reflect average differences between regional offices, type of assessment procedure, or time trends in application characteristics, disorder prevalence or clinical practice. We obtain the residuals from an ordinary‐least squares regression at the assessor level, with the leave‐one‐out leniency defined in (2) as dependent variable and regional office, type of assessment procedure and half‐year period as independent variables (Bakx et al., 2020; Dahl et al., 2014; Dobbie et al., 2018). Regional office and type of assessment procedure are included as dummies, while time is included as the proportion of applications assessed in each half‐year period. The distribution of residualized leniency is presented in Figure 1, together with the first‐stage results (discussed in Section 4.2.2.).

### 4.2 Two‐stage least squares regression approach

We use residualized leniency ($Leniency_{i}$) as an instrumental variable in a two‐stage least squares (2SLS) regression approach, to estimate the effects of becoming eligible to supported housing on the outcomes. Equation (3) provides the first‐stage regression while $\gamma_{1}$ in Equation (4) is the coefficient of interest.

(3) $$Eligibility_{i}=\beta_{0}+\beta_{1}Leniency_{i}+Control{s_{i}^{IV}}^{\prime }\beta^{C}+\varepsilon_{i}$$

(4) $$y_{i}=\gamma_{0}+\gamma_{1}{\hat{Eligibility}}_{i}+Control{s_{i}^{IV}}^{\prime }\gamma^{C}+n_{i}$$

The vector of $Controls_{i}^{IV}$ includes dummies for regional office, type of assessment procedure and half‐year periods, and an additional set of controls related to the applicant and the application discussed in more detail in Section 4.3.1.

#### 4.2.1 Interpretation

$\gamma_{1}$ represents the LATE for those individuals whose eligibility may be influenced by the assessor's residualized leniency: the compliers. While identification of the individual compliers is not feasible, we follow Dahl et al. (2014) and Dobbie et al. (2018) to estimate the proportion of marginal applicants in our study, and their characteristics as a group. Provided the monotonicity and independence assumptions hold, the share of always takers–those that would always be considered eligible regardless of assessor leniency–can be obtained from the eligibility decisions of the least lenient assessor. Analogously, the share of never‐takers–those that would never be granted eligibility no matter which assessor they are assigned to–can be derived from the decisions of the most lenient assessor. The group characteristics of the compliers are obtained by first defining subgroups based on characteristics potentially relevant for the assessor to utilize discretionary power. The relative likelihood that the compliers have these characteristics compared to the study population of applicants equals the first‐stage subgroup coefficient divided by the overall first‐stage coefficient (Maestas et al., 2013).

The LATE are meaningful from a policy perspective. Compliers are the individuals who might have received a different decision had their application been assigned to a different assessor. These individuals likely resemble those affected by marginal changes in future supported housing eligibility policy. Eligibility is a key policy tool for determining who should receive each type of long‐term mental health care. Hence, our findings are informative about access to the system rather than using supported housing, as individuals might not move into supported housing despite being eligible. For instance, individuals or the family might not accept the need to move into supported housing–as the application is often placed by a social worker or a provider–or they might not be willing to pay the co‐payments. Furthermore, eligible individuals usually only move into supported housing after some time because they need to find a suitable provider or make other arrangements. Conversely, those who are ineligible may re‐apply, for instance when their situation changes drastically, and become eligible and be admitted at a later point in time.

The group of compliers may change during the study period and we control for the half‐year period of the application to deal with any variation over time in either the population composition or assessor leniency, both within‐assessor or between early‐observation‐period and late‐observation‐period assessors. The effects we estimate should be interpreted as the weighted average over substantial treatment effect heterogeneity among the compliers with our instrument. Changes in composition and effect heterogeneity over time do not lead to biased estimators, but they do lead to a specific interpretation of the population of compliers which are represented by the compliers in our study.

#### 4.2.2 Relevance of the instrument

Leniency has a strong impact on eligibility: the share of individuals granted eligibility for supported housing ranges from 36% in the first percentile to 100% in the 99th percentile (average 86%, standard deviation 13%). The standard deviation of the residualized leniency measure is lower (9%), but still considerable. Figure 1 indicates a positive monotonic relationship between residualized leniency (*x*‐axis) and rate of eligibility to supported housing (*y*‐axis on the right). Consistent with Figure 1, the first‐stage coefficients indicate a strong and precisely estimated impact of residualized leniency on eligibility, which we observe regardless of the specification used (Table S2 of the appendix). Being assigned to a one standard deviation more lenient assessor increases the probability of being eligible for supported housing by 8.6% points (0.978 × 0.088) (Table S2). Furthermore, the first‐stage yields a Cragg‐Donald F‐statistic of 415.35.

#### 4.2.3 Validity

The Dutch institutional context, and in particular the allocation of applications to assessors, makes a violation of the instrument independence assumption unlikely.^8^ Applicants cannot choose the assessors, and assessors cannot choose the applications they review. Hence, assessors only interact with individuals and their families for the purpose of the needs‐assessment. Once the eligibility is granted, assessors have no other role in the pathway to utilization. Eligibility and provision of supported housing are taken care of by different parts of the Dutch long‐term care system: eligibility is decided upon by the independent needs‐assessment agency, while long‐term care is provided by private organizations that are contracted by a regional single payer. This means that the assessor's leniency should not be correlated with the outcomes of interest other than through the eligibility granted for supported housing. We report means of observed characteristics for each quartile of residualized leniency and find no indication of systematic or meaningful associations between such observed characteristics and residualized leniency (Tables S3 and S4 of the appendix).

#### 4.2.4 Monotonicity

In order to interpret our 2SLS estimates as LATE we must also assume average monotonicity, meaning that one application that is denied eligibility by the most lenient assessor would also be denied eligibility by the strictest assessor. Similarly, an application that is granted eligibility by the strictest assessor would also be granted eligibility by the most lenient one (Frandsen et al., 2019; Maestas et al., 2013). We do not have sufficient evidence to reject average monotonicity based on the positive relationship between residualized leniency and the probability of being eligible to supported housing in all major subgroups (Table 2, column 1) (Frandsen et al., 2019). We further confirm average monotonicity using alternative “reverse‐sample” instruments. These instruments are computed as the residualized leniency excluding the observations in the respective subgroup (e.g., we estimate the first‐stage for young adults based on assessor's residualized leniency constructed only with observations of mid‐age and late‐age adults) (Bhuller et al., 2019; Maestas et al., 2013). Our results show that assessors are stricter with a specific case type (i.e., the excluded subgroup) if they are also stricter with all other case types (Table 2, column 2).^9^

**TABLE 2:** First‐stage coefficients of residualized leniency for the entire study population and by subgroup

	Study population (main) (1)		Reverse‐sample (2)		Relative likelihood^c^ (3)	Observations (4)
	coefficient^a^	(se)	coefficient^b^	(se)		
Full population	0.978	(0.048)	n.a.	n.a.	1.00	7,953
Male	0.992	(0.050)	0.478	(0.075)	1.01	5,612
Female	0.935	(0.093)	0.682	(0.116)	0.95	2,341
Young adults (18–30)	1.152	(0.074)	0.848	(0.114)	1.18	3,250
Mid‐age adults (31–50)	0.964	(0.074)	0.662	(0.104)	0.99	2,898
Late‐age adults (51–79)	0.689	(0.107)	0.402	(0.093)	0.70	1,557
Dutch background	0.982	(0.056)	0.412	(0.065)	1.00	5,784
Western migration background	0.880	(0.157)	0.671	(0.174)	0.89	768
Non‐western migration background	0.968	(0.150)	0.666	(0.152)	0.98	1,401
Living alone	0.968	(0.072)	0.574	(0.092)	0.98	3,619
Living with partner/other	0.948	(0.143)	0.689	(0.140)	0.97	921
Living with parents	1.088	(0.099)	0.815	(0.132)	1.11	1,825
Living in an institution	0.863	(0.105)	0.515	(0.116)	0.88	1,588
Lowest quintile of income	1.111	(0.080)	0.934	(0.121)	1.13	1,595
Highest quintile of income	0.887	(0.099)	0.614	(0.104)	0.90	1,585
Prior work	1.135	(0.106)	0.904	(0.132)	1.16	2,027
Prior use of specialist mental health care	0.949	(0.056)	0.445	(0.083)	0.97	5,291
Prior use of home care	0.726	(0.101)	0.548	(0.101)	0.74	1,213
Lowest quintile of heath care expenditure	1.092	(0.113)	0.852	(0.125)	1.11	1,491
Highest quintile of health care expenditure	0.798	(0.121)	0.527	(0.131)	0.81	1,848
Substance use disorder	1.101	(0.102)	0.800	(0.124)	1.12	1,874
Psychotic disorder	0.570	(0.080)	0.391	(0.076)	0.58	1,531
Disorder diagnosed in the childhood	0.851	(0.128)	0.647	(0.149)	0.87	1,064
Mood disorder	0.902	(0.159)	0.693	(0.189)	0.92	663
Personality disorder	1.270	(0.167)	1.102	(0.192)	1.29	641
Other diagnosis	0.824	(0.147)	0.556	(0.139)	0.84	871
No diagnosis/treatment	1.175	(0.145)	0.656	(0.113)	1.20	1,350
Low intensity supported housing	0.866	(0.124)	0.650	(0.147)	0.88	659
Intermediate intensity supported housing	1.075	(0.078)	0.708	(0.097)	1.09	3,684
Intermediate‐high intensity supported housing	0.862	(0.081)	0.569	(0.094)	0.88	2,076
High intensity supported housing	0.931	(0.099)	0.666	(0.114)	0.95	1,534
Application by long‐term care provider	1.002	(0.056)	0.060	(0.061)	1.02	6,315
Application by social worker	0.909	(0.113)	0.665	(0.106)	0.92	1,084
Application by other^d^	0.885	(0.252)	0.133	(0.259)	0.90	554
Regular application	0.975	(0.050)	−0.21	(0.071)	0.99	7,671
Eligible for long‐term care last month^e^	0.949	(0.074)	0.694	(0.100)	0.97	2,708

*Note*: All regressions include the main specification controls, described in Section 4.3.1.

^a^ All first‐stage coefficients *p* < 0.001.

^b^ All first‐stage coefficients *p* < 0.001, except those of applications done by long‐term care providers or by others.

^c^ Relative likelihood is obtained by dividing subgroup‐specific first‐stage coefficients in column 1 by the full population first‐stage coefficient 0.978.

^d^ Including the candidate herself.

^e^ Except for supported housing.

### 4.3 Empirical implementation

We study five sets of outcomes: (a) *care use*: supported housing, home care and mental health care; (b) *expenditures*: both long‐term care expenditure (supported housing, home care and institutional care) and health care expenditure (mental health care–including outpatient and inpatient separately, and other medical non‐mental health care), which are summed in total expenditure; (c) *all‐cause mortality*; (d) *employment and income*: having a paid job, the amount of income from work, and total personal income^10^ and (*e*) *spill‐over effects on the parents*: mental health care use, having a paid job, income from work, total personal income. Parental outcomes are studied for the subpopulation for whom information about parents is available. Outcomes are studied in the calendar year after the application (all outcomes) and in the fourth calendar year after the application (mortality, employment, income, and parental outcomes).^11^

We include application‐related and individual‐related controls in the 2SLS regression. The application‐related controls are indicators for the regional office, type of assessment procedure (extended or abridged) and half‐year period; type of application (regular application or other routes such as priority cases) and the responsible for its submission (long‐term care provider, social worker or other); and whether  the applicant was eligible for other types of long‐term care in the last month. Individual‐level controls include age, gender, migration background (Dutch, western or non‐western first‐ or second‐generation migrant), living situation (alone, with partner, with parents, in an institution or other), prior use of specialist mental health care, prior health care expenditure, prior working and prior personal income (all for the calendar year before the application). Standard errors are clustered at the assessor level.

We explore subgroup effects of eligibility to supported housing on individual outcomes by age at the time of the application, prior personal income quintile, and mental disorder diagnosis. For parental outcomes, we consider heterogeneity for parents living with the child ahead of her application to supported housing. Robustness analyses include testing for the leniency instrumental variable approach, choices in the instrument construction, alternative 2SLS specifications and reporting findings on the individual outcomes for the subpopulation studied for parental spillovers.

## 5 RESULTS

We examine the effects of being eligible to supported housing using the variation in assessor's leniency as an instrument in a 2SLS regression approach. Results of the first‐stage show that assessors' leniency is highly predictive of whether an individual is deemed eligible to supported housing. An increase of 10 pp in the residualized instrument increases the probability of being eligible to supported housing by 9.8 pp (se: 0.5).

### 5.1 Individual outcomes

Being granted eligibility increased the probability of moving into supported housing by 31.6 pp (se: 5.7) in the calendar year after application (i.e., in the 12‐month period starting between 1 month‐application in December, and 11 months‐application in January, after the application) (Table 3). This effect shows that not every marginal individual granted eligibility will convert it into to admission during the following calendar year, and it compares with 17% of non‐eligible individuals that are admitted to supported housing in the same period, after re‐applying.^12^ Supported housing eligibility further decreased the likelihood of using home care by 13.4 pp (se: 6.4), relative to an average utilization of 30% among the non‐eligible. This is expected given that home care is an alternative form of support granted by assessors. We find no impact of supported housing eligibility on the likelihood of using mental health care and on all‐cause mortality.

**TABLE 3:** Two‐stage least squares (2SLS) regression estimates for the effects of being eligible for supported housing on individual outcomes

	Effect of eligibility for supported housing		First‐stage		Observations	Mean dependent variable non‐eligible group
	Coefficient	(se)	Coefficient	(se)		
**Calendar year after application**						
Supported housing admission	0.316***	(0.057)	0.978***	(0.048)	7,953	0.17
Use of home care	−0.134**	(0.064)	0.978***	(0.048)	7,953	0.30
Use of mental health care	−0.047	(0.05)	0.975***	(0.048)	7,852^a^	0.56
Total expenditure (€)	20,017***	(5,006)	0.978***	(0.048)	7,953	17,578
Supported housing expenditure (€)	11,883***	(2,634)	0.978***	(0.048)	7,953	4,850
Home care expenditure (€)	−280	(581)	0.978***	(0.048)	7,953	1,704
Medical expenditure (€)	−868	(824)	0.975***	(0.048)	7,852^a^	2,861
Total mental health care expenditure (€)	7,698*	(4,221)	0.975***	(0.048)	7,852^a^	6,440
Outpatient mental health care expenditure (€)	1,213*	(643)	0.975***	(0.048)	7,852^a^	2,419
Inpatient mental health care expenditure (€)	6,484	(4,183)	0.975***	(0.048)	7,852^a^	4,021
All‐cause mortality	−0.012	(0.014)	0.978***	(0.048)	7,953	0.01
Working	−0.070*	(0.036)	0.987***	(0.046)	7,596^a^	0.28
Income from work (€)	−2,149***	(717)	0.987***	(0.046)	7,596^a^	3,904
Personal income (€)	−1,470**	(748)	0.987***	(0.046)	7,596^a^	15,458
**Fourth calendar year after application**						
All‐cause mortality	0.05	(0.033)	0.978***	(0.048)	7,953	0.06
Working	−0.075	(0.055)	0.996***	(0.049)	7,218^a^	0.33
Income from work (€)	−3,410*	(1,488)	0.996***	(0.049)	7,218^a^	7,260
Personal income (€)	−2,017***	(747)	0.996***	(0.049)	7,218^a^	18,438

*Note*: Robust standard errors in parentheses, clustered at the assessor level; ***p < 0.01, **p < 0.05, *p < 0.1.

^a^ Fewer observations due to missing data in the health insurance claims and tax returns databases. The analysis was replicated for the smallest sample available for all outcomes –results available per request. All regressions include the main specification controls described in Section 4.3.

Being eligible for supported housing increased total care spending with 20,017 euros (se: 5,006) in the following calendar year (average spending among the non‐eligible of 17,578 euros). This is mostly due to the higher costs of supported housing (11,883 euros, se: 2,634) but also due to higher mental health care expenditure, for which the coefficient is less precisely estimated but large in magnitude (7,698 euros, se: 4,221). Findings on mental health care expenditure reported separately for the outpatient and inpatient settings suggest a higher consumption of outpatient care (1,213 euros, se: 643) by those eligible.

Income from work (unconditional on working) is 2,149 euros (se: 717) lower, which is large compared to the non‐eligible average of 3,904 euros. This is in line with results suggesting lower likelihood of in being in paid employment by 7.0 pp (se: 3.6), which corresponds to a 25% reduction compared to the non‐eligible group (−7.0/28.0). Total personal income decreased by an average of 1,470 euros (se: 748; average among non‐eligible 15,458 euros) indicating that the income loss from work was partly compensated by other sources of income. (Income from) paid employment captures regular (competitive) jobs and sheltered employment, which offers individuals with a disability a protected environment with extra guidance and employees get paid at least the minimum wage. After 4 years, labor related effects are consistently more negative in absolute terms.

The 2SLS results in Table 3 provide consistent estimates of the average treatment effects for the group of compliers (LATE). Ordinary‐least squares estimates would, in theory, capture the average treatment effects for the population, but are very likely biased (Table S6 of the appendix). A better understanding of the LATE can be achieved with further characterization of the compliers. The compliers comprise 41% of the population and the remaining individuals are always‐takers. The absence of never‐takers possibly reflects the nature of the intervention, the institutional setting, and the selection of the study population. As compared to the full study population, the relative likelihood that the compliers have certain characteristics is presented in column 3 of Table 2. Compliers are more likely than the average individual to be young adults, have worked in the prior year, to have a personality disorder or no treatment/diagnosis of mental disorder (in the previous 365 days); and less likely to be late‐age adults, to live with a partner at the time of the application, to have prior home care use or high prior health care expenditure, have a psychotic disorder or to be in the category other diagnosis.

### 5.2 Parental outcomes

Table 4 displays the effects on parental mental health care use and labour outcomes for the subpopulation for whom information about parents is available. Information about at least one parental outcome is available for 72% of the individuals in the full study population, ranging from 48% to 66% depending on the outcome.^13^ This group differs from the study population by being on average younger, more likely to live with the parents, work ahead of the application, and to have a disorder diagnosed during childhood (Table S7 of appendix). Overall, effects of supported housing eligibility on individual outcomes among those with information on at least one parental outcome are qualitatively and statistically consistent with those in the study population, although the magnitude of the effects is smaller for supported housing admission and more precisely estimated for a decrease in the individual labor participation in the short‐run (Table S8 of the appendix).

**TABLE 4:** Two‐stage least squares (2SLS) regression estimates for the effects of being eligible for supported housing on parental outcomes

		Effect of eligibility for supported housing		First‐stage		Observations	Mean dependent variable non‐eligible group
		Coefficient	(se)	Coefficient	(se)		
**Calendar year after application**							
**Mother**	Use of mental health care	−0.010	(0.059)	1.186***	(0.053)	5,240	0.12
	Working	0.068	(0.044)	1.197***	(0.052)	5,097	0.40
	Income from work (€)	2,582	(1676)	1.197***	(0.052)	5,097	9,749
	Personal income (€)	1,620	(1160)	1.197***	(0.052)	5,097	17,161
**Father**	Use of mental health care	0.046	(0.043)	1.085***	(0.076)	4,169	0.08
	Working	0.094	(0.06)	1.128***	(0.080)	4,061	0.54
	Income from work (€)	1,951	(4,185)	1.128***	(0.080)	4,061	25,877
	Personal income (€)	489	(3,304)	1.128***	(0.080)	4,061	34,651
**Fourth calendar year after application**							
**Mother**	Working	0.151**	(0.072)	1.105***	(0.053)	4,835	0.36
	Income from work (€)	1,866	(2,347)	1.105***	(0.053)	4,835	9,779
	Personal income (€)	−98	(1,332)	1.105***	(0.053)	4,835	18,629
**Father**	Working	0.178**	(0.076)	1.102***	(0.078)	3,765	0.51
	Income from work (€)	1,957	(4,679)	1.102***	(0.078)	3,765	27,799
	Personal income (€)	909	(4,065)	1.102***	(0.078)	3,765	36,505

*Note*: Robust standard errors in parentheses, clustered at the assessor level; ***p < 0.01, **p < 0.05, *p < 0.1. All regressions include the main specification controls described in Section 4.3.

Over time, child eligibility for supported housing progressively increases the likelihood of parents being in a paid job, and leads to 15.1 pp (se: 7.2 − mothers) and 17.8 pp (se: 7.6 − fathers) increases on the forth calendar year after the application. The relative size of these effects is larger for the mothers, for which the average population employment is lower than for fathers (36% vs. 51%). No precise estimates are found for the other parental outcomes.

### 5.3 Subgroup analysis

We study subgroup effects by age, personal income quintile and mental disorder diagnosis, which are relevant characteristic for potential heterogenous effects. First, different age groups (18–30, 31–50 and 51–79 years old) correspond to different profiles in terms of living situation and social network. While the youngest group often applies for supported housing leaving behind their family house, the older age groups tend to have a much more reduced social network (living on their own, in other institutions or be homeless), with different potential effects on social functioning and parental outcomes. Second, social disadvantage (proxied by the lower personal income quintiles) likely increases the effect of eligibility on admission to supported housing, while individuals in higher income quintiles are better surrounded by (in)formal caregiving and support at home. Last, the type of disorder (substance use disorder, psychotic disorder, disorder diagnosed in the childhood, mood disorder, personality disorder, other disorder and no treatment/diagnosis) may influence the extent to which individuals may recover and live an independent life and work.

**FIGURE 2:**
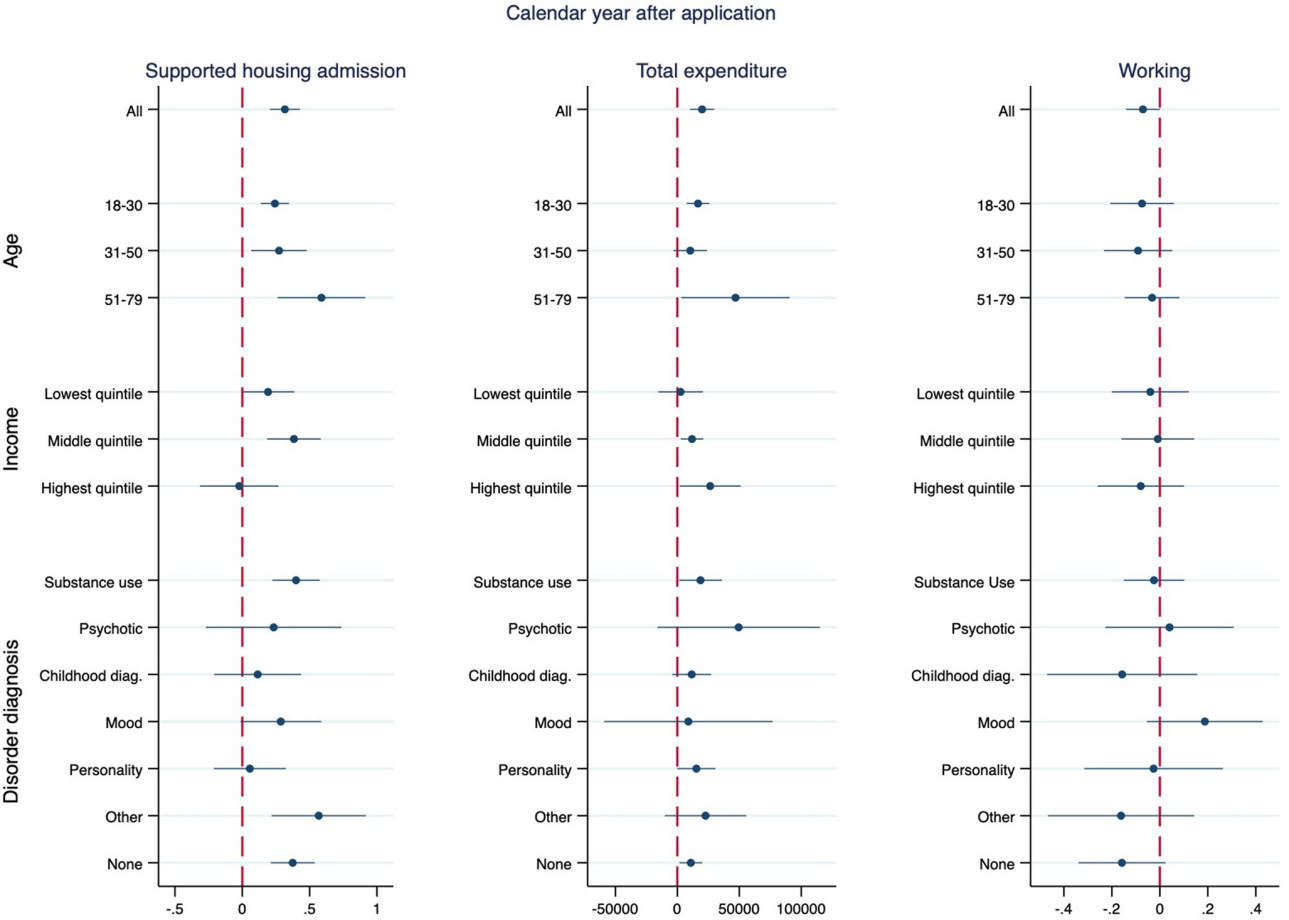
Subgroup analysis for admission to supported housing, total expenditure and working in the next calendar year: by age, personal income quintile and mental disorder diagnosis

Our findings for subgroup effects show limited heterogeneity for most outcomes, even though mostly imprecise (Figure 2 and Figure S1 in the appendix). Point estimates are suggestive of potential heterogenous effects in admission to supported housing in the next calendar year (for older individuals and those with a personality disorder) and in the likelihood of working in the short‐run (for those with mood disorders). The spillovers effects among parents living with the child ahead of her application confirm the patterns found in the main analyses for the parental outcomes, but the effects are much less precisely estimated (Table S9 of the appendix).

### 5.4 Robustness analysis

We test the robustness of our main specification in five ways. First, results are robust to the stepwise inclusion of different sets of individual/application controls in the main specification, as well as controlling for age as dummies and additional controls for mental disorder diagnosis and need at the time of application, as proxied by the care intensity requested (columns 2–6 in Table S2 of the appendix). Second, results are also robust to changing the minimum number of cases by assessor/type of assessment procedure from 30 to 15 or to 45, when constructing the leniency instrument (columns 7–8 in Table S2). Third, the effect size is slightly reduced after including all the applications likely to be reassessments of recent eligibility status, by removing the requirement that individuals had not been eligible for supported housing in the past 365 days (column 9 in Table S2). Fourth, we find similar results when using the unadjusted version of the leniency measure (column 10 in Table S2) and when residualizing leniency by quarter of the year (Table S10 of the appendix). Last, we replace the residualized leniency by a vector of assessor fixed effects, using the unbiased jackknife instrumental variables estimator (UJIVE) (Kolesar, 2013). Following Bakx et al. (2020), Dahl et al. (2014), and Maestas et al. (2013), we have opted for the residualized leave‐one‐out leniency instrument in our main specification; but Hull (2017) argues that the implied dimension reduction procedure of the assessor fixed effects instruments may result in an overidentified first‐stage. The many weak instruments problem could be masked by the use of the leniency, which is a continuous instrument composed of the assessor‐level averages of the endogenous variable. Concerns are that, as the dimensionality of the underlying variation is not one but the number of assessor fixed effects, first‐stage F‐statistics might overstate the instrument strength (Hull, 2017). To investigate this concern we estimate our results using UJIVE,^14^ a 2SLS estimator that is “consistent for a convex combination of LATE under many instrument asymptotics (and) that also allow for many covariates and heterocedasticity” (Kolesar, 2013). Results obtained using UJIVE confirm that the main results are unlikely strongly biased due to weak instruments (Tables S11 and S12 of the appendix).

## 6 DISCUSSION

This paper studies the impact of supported housing eligibility for people with mental disorders on care use, mortality, employment and income, and on spillovers to their parents. We report four main findings. First, compliers who are being assigned to a more lenient assessor are more likely granted eligibility to supported housing. This, in turn, increases the probability of an admission to supported housing and reduces the likelihood of getting home care. Second, public care spending increases mainly via higher expenditures on supported housing. Findings do also suggest increased total mental health care expenditure and specifically higher outpatient mental health care expenditure. One potential mechanism behind this finding could be that in more supervised environments individuals increase their adherence to medical appointments, psychotherapy or medication. The effect on inpatient mental care is not significant, suggesting that supported housing does not considerably prevent inpatient admissions. Finally, expenditures on other types of care do not change.

Third, eligibility for supported housing decreases income from work and personal income, and likely the probability of having paid work. Fourth, both parents are more likely to work in the long‐run, and these effects are stronger among parents living with their child before application. While the spillover effects are likely explained by the fact that paid work and informal care‐giving are substitutes, the mechanisms behind lower employment participation and earnings are for the applicants are less obvious. Labor participation determinants for individuals with severe mental illness include personal factors such as self‐efficacy and self‐stigma, or work related factors such as employers stigma and discrimination (Van Weeghel & Michon, 2018); which might impact supported housing clients differently than individuals remaining at home. Individuals might feel permanently disabled by their illness (defeatism), not only due to the actual disability, but also due to negative experiences in the workforce, or the process of applying and then becoming locked into disability benefits (Drake et al., 2013). On the other hand, mental health practitioners may be ambivalent about the value of work for the group that becomes eligible, even though occupational rehabilitation practices have been changing toward placing individuals in competitive employment (Van Weeghel & Michon, 2018). The larger drop in income from work than in total personal income (which includes transfers) suggests that individuals in supported housing apply for social security more often, which might be explained by increased financial counseling or different eligibility due to changes in their household composition.

Our findings should be interpreted having in mind three considerations. First, the group of applicants that are granted eligibility to supported housing is composed of a mix of individuals with differences in key characteristics such as age, prior living situation and mental disorder diagnosis. A fifth of our population is between 18 and 21 years old, a subgroup that differs in terms of their higher likelihood of previously working, living with parents ahead of the application and having a disorder diagnosed during childhood or other or no diagnosis (Table S13 of the appendix). An additional fifth of the population is between 22 and 30 years old, leading to an overall total of 40% that is aged 30 or less. Supported housing clients in the Netherlands also differ in terms of their care needs and recovery possibility (Bitter et al., 2016). In our population, the different durations of the supported housing eligibility indications is a sign of this heterogeneity: 54% of the individuals are granted 3 years or less, indicating that they are expected to return to living independently/in the community in the short‐medium term; and 32% of the individuals are granted the maximum duration of supported housing possible–15 years. The latter individuals need a high level of living support for a long time, and improvements in employment or autonomy are less probably. Our findings for subgroup estimates are imprecise, possibly due to opposite effects canceling out and not all recovery profiles being represented among the compliers, but do suggest that admission to supported housing depends on age and disorder type.

Second, it is important to reflect on the care received by the non‐eligible group. Fifty‐three percent of the individuals who are not granted eligibility for supported housing are eligible for home care, while another 41% are granted no long‐term care at all. Individuals judged as needing different types of alternatives to supported housing are likely to have different profiles and background characteristics such as more access to informal care and a wider support network. It is possible that there are heterogeneous effects within our control group that we cannot disentangle, namely that having some formal home care (individual or group guidance provided by floating outreach staff that visit individuals in their own tenancies) has a different impact than relying only on informal care. Nevertheless, we observe from the average spending on home care that the intensity of guidance being provided is limited,^15^ and should not represent a large difference in terms of support for these subgroups. The remaining 6% of non‐eligible individuals was granted different forms of institutional care such as nursing homes or institutions for disabled: our checks indicate that this small group (<1% study population) should have no influence on our results (Table S14 and Figures S2 to S4 of the appendix).

Last, it is important to acknowledge the limitations of our data in capturing some relevant dimensions. First, administrative data does not fully capture individual level benefits of supported housing in terms of well‐being and health‐related quality of life and housing retention. Second, paid employment might also be considered as limited to capture social participation: it does not capture voluntary work, and does not allow distinguishing those employed in competitive (regular) jobs from those in sheltered employment. It is not clear if being in sheltered employment would affect the probability of work, or the amount of earnings. It might be worth noticing that both voluntary arrangements and sheltered employment were more prominent at the time of our study than in the present days, given the current focus of programs such as Individual Placement and Support to place individuals in supported housing in competitive employment (Roeg et al., 2021).

Overall, our results seem to support some of the criticisms toward supported housing: that it is costly form of care and might undermine patient's social functioning (Chilvers et al., 2006; Fakhoury & Priebe, 2007), which we document through labor market outcomes. On the other hand, the results suggest positive spillovers to parental employment. While we are only able to measure a subset of relevant outcomes, our study suggests that there is a trade‐off to be considered between different effects. Further policy decisions on extending supervised environments should deal with such trade‐offs by weighting the effects in the various domains and perspectives–the system, the individuals and their families.

## CONFLICT OF INTEREST

The authors declare no conflict of interest.

## Supporting information

Supplementary Material

## Data Availability

This project uses non‐public microdata provided by Statistics Netherlands together with data made available to the authors by CIZ, both accessed via Statistics Netherlands remote access facility. Under certain conditions, Statistics Netherlands microdata are accessible for statistical and scientific research. Applications should be made through a government data sharing portal (https://www.cbs.nl/en‐gb/our‐services/customised‐services‐microdata/microdata‐conducting‐your‐own‐research; for further information contact microdata@cbs.nl).

## ENDNOTES

^1^
Other designations use for supported housing include protected living, sheltered living, or supportive housing.

^2^
Individual or group guidance is provided according to practices of floating outreach support or supported independent living, at the individual's own home.

^3^
Back‐office employees handle delegated reassessments (*Herindicatie* via *taakmandaat*–HIT) and applications for types of care for which a predefined procedure is available (*Standaard Indicatieprotocol*–SIP), which are often directly approved. A small number of these application might be checked by assessors: HIT would be checked before the approval is granted and SIP afterward.

^4^
Long‐term care applications to CIZ could also be assessed through a standard assessment procedure. This procedure is similar to the abridged assessment with an additional a face‐to‐face interview. The number of standard assessments in our population was residual and hence these were excluded (Table S1 in the appendix).

^5^
The latter group of applications correspond either to requests to extend eligibility or to changes between different intensities of support for those potentially using it, or to re‐assessments post‐termination within a short time frame. In all these cases, the assessor's leniency will be considerably influenced by the previous assessment.

^6^
The instrument is estimated based on data coming solely from the CIZ dataset of all long‐term applications, available from 2009 to 2013. In the remaining administrative datasets several outcomes and controls of interest could not be measured for 2009 and 2010, namely the actual use of supported housing (2009 and 2010), and mental health diagnosis and health care expenditures in the year prior to the application (2009).

^7^
Disorders diagnosed during childhood most commonly refer to pervasive and specific developmental disorders such as autistic disorders, Asperger's disorder, and behavioral and emotional disorders such as attention deficit hyperactivity disorders, and conduct disorders. 18% of the applications had no specialist mental health treatment in the previous 365 days, so there was no information about the mental disorder diagnosis.

^8^
The Dutch government announcement in 2012 to reduce the number of inpatient mental health treatments (Trimbos‐instituut, 2021) –which is in line with the ongoing trend of deinstitutionalization (Chow & Priebe, 2016; Fakhoury & Priebe, 2007; Ravelli, 2006; Thornicroft et al., 2016)– could pose a threat to the identification of the LATE. Our approach of controlling for half‐year periods when constructing the residualized instrument and when applying the 2SLS estimates accounts for these changes. In the same way, we account for regional offices tackling the different nature of the applications by geography.

^9^
Monotonicity at the individual‐level (strict monotonicity) is a requirement to estimate marginal treatment effects (MTE). We use the test of Frandsen et al. (2019), that jointly checks for the exclusion and strict monotonicity assumptions. As suggested by the authors, and given that our institutional context supports excludability, we interpret Frandsen's test as a test for strict monotonicity only. The estimated test statistics indicate a violation of strict monotonicity (Table S5 of the appendix). This prevents estimation of MTE, but has no implications on the average monotonicity condition, under which the instrumental variables estimator converges to a proper weighted average of the LATE.

^10^
Income and health care expenditures are expressed in prices of 2015 using a Consumer Price Index (CBS, 2022).

^11^
Expenditures are only studied in the short‐term due to changes in the data available after the reform of 2015, which shifted funding and organization of supported housing and home care to the municipality level (see also Section 3.1).

^12^
The estimate in Table 3 gives the effect on admission in the next calendar year. Admission into supported housing in the 90, 180 and 365 days following application increased by respectively 33.8 pp, 35.2 pp, and 38.6 pp. The increasing trend indicates that the share converting eligibility into admission rises more strongly over time for the eligible compared to the non‐eligible that get admitted after re‐applying.

^13^
There are several reasons for parental outcomes not being available. On the one hand, the identity of the father and the mother might not be known (29% and 17% of all missing observations for parents, respectively): parents might have never lived in the Netherlands and child‐parent linkages are only available for parents alive from 1995. On the other hand, outcome data might be missing for parents observed in the data: parents have moved abroad or have died, either before the application was placed or between the application and the measurement of outcomes. Importantly, there is no missing data for parental outcomes in the subgroup of individuals living with their parents at the time for the application, for which we conduct a robustness analysis.

^14^
UJIVE was proposed by Kólesar (2013) as an alternative to the jackknife instrumental variables estimator (JIVE) (Angrist et al., 1999). UJIVE is similar to JIVE in that it “also uses a “leave‐one‐out” jackknife‐type predictor of the treatment in the first‐stage”, but it differs from the latter in the way it deals with the covariates, as JIVE is not robust to a large number of covariates. The UJIVE partials out the effect of the covariates on the treatment, guaranteeing that the prediction error will be uncorrelated with the outcome.

^15^
Considering the average hourly cost of 58 euros for home care (weighted average between individual guidance‐80%, group guidance‐15% and nursing‐5%), the average consumption of home care in the next calendar year is 26 min/week (1,513 euros/58 euros/52 weeks).
