# Supplementary material for: The effects of supported housing for individuals with mental disorders
Source: Health Econ. 2022 Aug 19;31(Suppl 2):115–33. doi: 10.1002/hec.4579 (PMC9805038; doi:10.1002/hec.4579)
Supplement: Supplementary file 1 — Supplementary Material [file HEC-31-115-s001.docx]

**The effects of supported housing for individuals with mental disorders**

**Online Appendix**

Table S1. Selection of the study population

|  | % of applications to supported housing 2009-2013 † | Number of observations left following exclusion | Exclusion criteria |
| --- | --- | --- | --- |
| Applications to supported housing 2009-2013 | 100.0% | 34,107 |  |
| Information on regional office missing | 0.1% | 34,078 | Needed to estimate leniency |
| Information on assessor missing ‡ | 4.1% | 32,682 | Needed to estimate leniency |
| Age <18 | 0,1% | 32,638 | Not part of the population of interest |
| Age > 79 | 3.6% | 31,423 | Margin between supported housing and institutional care |
| Psychogeriatric indication | 0.5% | 31,244 |  |
| Intellectual, sensory or physical disability indication | 6.4% | 29,072 |  |
| Palliative care | <0.1% | 29,059 |  |
| Backoffice decision § | <0.1% | 29,052 | Not relevant for leniency (limited discretionary power) |
| Valid supported housing eligibility decision < 365 days | 27.0% | 19,828 |  |
| Emergency applications | 0.9% | 19,522 |  |
| Applications following pre-defined routes | 1.3% | 19,086 |  |
| Standard assessment | 0.7% | 18,839 | Insufficient applications per assessor for leniency estimation |
| < 30 applications assessor/assessment/regional office | 17.8% | 12,767 | To allow minimum precision in the estimate of leniency |
| 2009 and 2010 or missing data on controls | 14.1% | 7,953 |  |

† Criteria are not mutually exclusive. ‡ Most applications without information on assessor correspond to backoffice decisions. § Backoffice decisions with an assessor number might correspond to SIP (predefined procedure) or HIT (delegated reassessments) applications that were checked by assessors.

Table S2. Two-stage least squares (2SLS) regression estimates for different specifications on the outcome admission to supported housing.

|  | Main model | | Different set of controls used in the 2SLS with preferred measure of leniency (residuals) | | | | | | Minimum number of cases by assessor † | | Including reassessments ‡ | | | Unadjustedleniency | |  |
| --- | --- | --- | --- | --- | --- | --- | --- | --- | --- | --- | --- | --- | --- | --- | --- | --- |
|  | (1) | (2) | | (3) | (4) | (5) | (6) | (7) | | (8) | | (9) | (10) | |  |  |
|  |  |  | |  |  |  |  |  | |  | |  |  | |  |  |
| Effects of eligibility on supported housing admission (se) | 0.316*** | 0.328*** | | 0.349*** | 0.333*** | 0.333*** | 0.305*** | 0.346*** | | 0.343*** | | 0.301*** | 0.319*** | |  |  |
|  | (0.057) | (0.067) | | (0.560) | (0.055) | (0.055) | (0.057) | (0.049) | | (0.077) | | (0.055) | (0.068) | |  |  |
|  |  |  | |  |  |  |  |  | |  | |  |  | |  |  |
| First-stage (se)  Effect of leniency on eligibility | 0.978*** | 1.132*** | | 0.993*** | 0.980*** | 0.976*** | 0.965*** | 0.971*** | | 0.946*** | | 0.958*** | 0.790*** | |  |  |
|  | (0.048) | (0.123) | | (0.048) | (0.048) | (0.048) | (0.049) | (0.040) | | (0.084) | | (0.048) | (0.054) | |  |  |
|  |  |  | |  |  |  |  |  | |  | |  |  | |  |  |
| Controlling for: |  |  | |  |  |  |  |  | |  | |  |  | |  |  |
| Region, period and type of assessment | x |  | | x | x | x | x | x | | x | | x | x | |  |  |
| Patient characteristics | x |  | |  | x | x (age dummies) | x | x | | x | | x | x | |  |  |
| Application characteristics | x |  | |  |  |  | x | x | | x | | x | x | |  |  |
| Diagnosis and care requested |  |  | |  |  |  | x |  | |  | |  |  | |  |  |
|  |  |  | |  |  |  |  |  | |  | |  |  | |  |  |
| Minimum number of cases/assessor | 30 | 30 | | 30 | 30 | 30 | 30 | 15 | | 45 | | 30 | 30 | |  |  |
|  |  |  | |  |  |  |  |  | |  | |  |  | |  |  |
| Observations | 7,953 | 7,953 | | 7,953 | 7,953 | 7,953 | 7,953 | 9,362 | | 6,633 | | 11,459 | 7,953 | |  |  |
| F-statistic (p-value) for weak identification (Cragg-Donald) | 415.35 (0.000) | 84.8 (0.000) | | 429.4 (0.000) | 421.7 (0.000) | 416.4 (0.000) | 392.9  (0.000) | 591.3  (0.000) | | 126.0 (0.000) | | 404.0  (0.000) | 211.8  (0.000) | |  |  |
| Robust standard errors in parentheses, clustered at the assessor level; *** p<0.01, ** p<0.05, * p<0.1. † minimum number of cases by assessor, region and type of assessment ‡Applications with a valid eligibility decision to supported housing in the 365 days before the application. Results for all other outcomes are available per request for robustness checks done in columns (6) to (10): estimates are consistent with our main findings. | | | | | | | | | | | | | | | | |

| Table S3. Association between observables and leniency: covariate means by leniency quartile and ANOVA | | | | | | | |
| --- | --- | --- | --- | --- | --- | --- | --- |
|  | Total | 1st quart | 2nd quart | 3rd quart | 4th quart | ANOVA F | Prob > F † |
|  | 7,953 | 2,030 | 1,956 | 2,001 | 1,966 |  |  |
| Female | 0.29 | 0.30 | 0.29 | 0.27 | 0.32 | 3.637 | 0.0123 |
| Age | 37.73 | 38.17 | 38.25 | 37.09 | 37.39 | 2.717 | 0.0430 |
| Dutch background | 0.73 | 0.72 | 0.74 | 0.72 | 0.72 | 0.742 | 0.5267 |
| Western background | 0.10 | 0.10 | 0.09 | 0.09 | 0.10 | 0.664 | 0.5744 |
| Non-Western background | 0.18 | 0.17 | 0.17 | 0.19 | 0.18 | 1.114 | 0.3417 |
| Living alone | 0.46 | 0.44 | 0.46 | 0.46 | 0.46 | 0.490 | 0.6895 |
| Living with partner | 0.07 | 0.08 | 0.07 | 0.07 | 0.07 | 0.721 | 0.5393 |
| Living with parents | 0.15 | 0.14 | 0.15 | 0.16 | 0.14 | 0.823 | 0.4812 |
| Living with single parent | 0.08 | 0.08 | 0.07 | 0.08 | 0.08 | 1.423 | 0.2338 |
| Living institution | 0.20 | 0.20 | 0.21 | 0.18 | 0.20 | 2.168 | 0.0896 |
| Other in household | 0.05 | 0.05 | 0.05 | 0.04 | 0.04 | 1.529 | 0.2046 |
| Prior personal income, 1st quintile | 0.20 | 0.20 | 0.20 | 0.19 | 0.22 | 1.760 | 0.1525 |
| Prior personal income, 5^th^ quintile | 0.20 | 0.20 | 0.19 | 0.20 | 0.21 | 0.647 | 0.5847 |
| Prior working | 0.25 | 0.26 | 0.24 | 0.26 | 0.25 | 1.082 | 0.3552 |
| Prior specialist mental health care | 0.67 | 0.65 | 0.66 | 0.69 | 0.66 | 3.020 | 0.0286 |
| Prior home care | 0.15 | 0.15 | 0.16 | 0.15 | 0.15 | 0.691 | 0.5574 |
| Prior health care exp., 1^st^ quintile | 0.19 | 0.19 | 0.19 | 0.18 | 0.19 | 0.674 | 0.5679 |
| Prior health care exp., 5^th^ quintile | 0.23 | 0.22 | 0.22 | 0.25 | 0.24 | 2.072 | 0.1017 |
| Substance use disorder | 0.24 | 0.22 | 0.24 | 0.25 | 0.23 | 2.216 | 0.0841 |
| Psychotic disorder | 0.19 | 0.18 | 0.19 | 0.20 | 0.20 | 0.716 | 0.5423 |
| Disorder diagnosed in the childhood | 0.13 | 0.12 | 0.13 | 0.14 | 0.14 | 1.025 | 0.3803 |
| Mood disorder | 0.08 | 0.08 | 0.08 | 0.08 | 0.08 | 0.014 | 0.9978 |
| Personality disorder | 0.08 | 0.09 | 0.08 | 0.07 | 0.08 | 0.956 | 0.4126 |
| Other diagnosis | 0.10 | 0.10 | 0.09 | 0.09 | 0.10 | 0.808 | 0.4894 |
| No treatment/diagnosis | 0.18 | 0.20 | 0.18 | 0.16 | 0.17 | 4.930 | 0.0020 |
| Low intensity suppported housing | 0.08 | 0.10 | 0.08 | 0.07 | 0.08 | 3.736 | 0.0107 |
| Intermediate intensity supported housing | 0.46 | 0.47 | 0.45 | 0.49 | 0.45 | 3.719 | 0.0109 |
| Intermediate-high intensity supported housing | 0.26 | 0.25 | 0.28 | 0.25 | 0.26 | 2.465 | 0.0604 |
| High intensity supported housing | 0.19 | 0.18 | 0.19 | 0.19 | 0.21 | 1.180 | 0.3157 |
| Application by long-term care provider | 0.79 | 0.78 | 0.80 | 0.80 | 0.80 | 0.809 | 0.4885 |
| Application by social worker | 0.07 | 0.06 | 0.07 | 0.08 | 0.07 | 1.782 | 0.1483 |
| Application by other ‡ | 0.14 | 0.16 | 0.13 | 0.13 | 0.13 | 4.050 | 0.0069 |
| Regular application | 0.96 | 0.97 | 0.96 | 0.95 | 0.97 | 5.562 | 0.0008 |
| Eligible for long-term care last month§ | 0.34 | 0.35 | 0.35 | 0.33 | 0.33 | 1.368 | 0.2506 |

† When corrected for multiple testing using the Bonferroni correction, Prob > F is larger than 0.01 for all observed characteristics. ‡ Including the candidate herself § Except for supported housing as all those eligible for supported housing in the 365 days ahead of the application were excluded.

Table S4. Association between observables and leniency: covariate means by leniency quartile and t-test vs. 1^st^ quartile

|  | Total  7,953 | 1st quart  2,030 | 2nd quart  1,956 | 3rd quart  2,001 | 4th quart  1,966 | 2nd quart vs. 1st quart  t-test | 3rd quart vs. 1st quart  t-test | 4th quart vs. 1st quart  t-test |
| --- | --- | --- | --- | --- | --- | --- | --- | --- |
| Female | 0.29 | 0.30 | 0.29 | 0.27 | 0.32 | 0.501 | 0.083 | 0.134 |
| Age | 37.73 | 38.17 | 38.25 | 37.09 | 37.39 | 0.873 | 0.027 | 0.116 |
| Dutch background | 0.73 | 0.72 | 0.74 | 0.72 | 0.72 | 0.236 | 0.915 | 0.980 |
| Western background | 0.10 | 0.10 | 0.09 | 0.09 | 0.10 | 0.321 | 0.181 | 0.543 |
| Non-Western background | 0.18 | 0.17 | 0.17 | 0.19 | 0.18 | 0.541 | 0.247 | 0.611 |
| Living alone | 0.46 | 0.44 | 0.46 | 0.46 | 0.46 | 0.420 | 0.266 | 0.342 |
| Living with parents | 0.23 | 0.23 | 0.22 | 0.24 | 0.23 | 0.504 | 0.184 | 0.863 |
| Living institution | 0.20 | 0.20 | 0.21 | 0.18 | 0.20 | 0.304 | 0.134 | 0.847 |
| Living with partner/other household | 0.12 | 0.13 | 0.11 | 0.11 | 0.11 | 0.098 | 0.106 | 0.055 |
| Prior personal income, 1st quintile | 0.20 | 0.20 | 0.20 | 0.19 | 0.22 | 0.865 | 0.332 | 0.194 |
| Prior personal income, 5th quintile | 0.20 | 0.20 | 0.19 | 0.20 | 0.21 | 0.671 | 0.851 | 0.345 |
| Prior working | 0.25 | 0.26 | 0.24 | 0.26 | 0.25 | 0.152 | 0.925 | 0.354 |
| Prior specialist mental health care | 0.67 | 0.65 | 0.66 | 0.69 | 0.66 | 0.252 | 0.003 | 0.220 |
| Prior home care | 0.15 | 0.15 | 0.16 | 0.15 | 0.15 | 0.247 | 0.918 | 0.912 |
| Prior health care exp., 1st quintile | 0.19 | 0.19 | 0.19 | 0.18 | 0.19 | 0.882 | 0.201 | 0.471 |
| Prior health care exp., 5^th^ quintile | 0.23 | 0.22 | 0.22 | 0.25 | 0.24 | 0.693 | 0.041 | 0.067 |
| Substance use disorder | 0.24 | 0.22 | 0.24 | 0.25 | 0.23 | 0.055 | 0.018 | 0.336 |
| Psychotic disorder | 0.19 | 0.18 | 0.19 | 0.20 | 0.20 | 0.494 | 0.269 | 0.168 |
| Disorder diagnosed in the childhood | 0.13 | 0.12 | 0.13 | 0.14 | 0.14 | 0.587 | 0.127 | 0.169 |
| Mood disorder | 0.08 | 0.08 | 0.08 | 0.08 | 0.08 | 0.918 | 0.838 | 0.926 |
| Personality disorder | 0.08 | 0.09 | 0.08 | 0.07 | 0.08 | 0.968 | 0.133 | 0.538 |
| Other diagnosis | 0.10 | 0.10 | 0.09 | 0.09 | 0.10 | 0.194 | 0.518 | 0.939 |
| No treatment/diagnosis | 0.18 | 0.20 | 0.18 | 0.16 | 0.17 | 0.033 | 0.001 | 0.001 |
| Low intensity supported housing | 0.08 | 0.10 | 0.08 | 0.07 | 0.08 | 0.049 | 0.001 | 0.167 |
| Intermediate intensity supported housing | 0.46 | 0.47 | 0.45 | 0.49 | 0.45 | 0.080 | 0.314 | 0.078 |
| Intermediate-high intensity supported housing | 0.26 | 0.25 | 0.28 | 0.25 | 0.26 | 0.011 | 0.630 | 0.200 |
| High intensity supported housing | 0.19 | 0.18 | 0.19 | 0.19 | 0.21 | 0.419 | 0.616 | 0.069 |
| Application by long-term care provider | 0.96 | 0.97 | 0.96 | 0.95 | 0.97 | 0.273 | 0.003 | 0.381 |
| Application by social worker | 0.79 | 0.78 | 0.80 | 0.80 | 0.80 | 0.270 | 0.248 | 0.154 |
| Application by other † | 0.14 | 0.16 | 0.13 | 0.13 | 0.13 | 0.010 | 0.004 | 0.005 |
| Regular application | 0.07 | 0.06 | 0.07 | 0.08 | 0.07 | 0.065 | 0.032 | 0.093 |
| Eligible for long-term care last month ‡ | 0.34 | 0.35 | 0.35 | 0.33 | 0.33 | 0.947 | 0.134 | 0.150 |

† Including the candidate herself ‡ Except for supported accommodation as all those eligible for supported accommodation in the 365 days ahead of the application were excluded.

Table S5. Joint test for exclusion and strict monotonicity assumptions (Frandsen et al. 2019)

|  | Individual outcomes | | | | | Parental outcomes (mother/father) | | |  |
| --- | --- | --- | --- | --- | --- | --- | --- | --- | --- |
|  | 1^st^ year | | 4^th^ year | 1^st^ year | | | | 4^th^ year |  |
| Outcome | p-value | p-value | | | p-value | | p-value | | |
| Supported accommodation admission | 0.137 | n.a. | | | n.a. | | n.a. | | |
| Use of home care | 0.055 | n.a. | | | n.a. | | n.a. | | |
| Use of mental health care | 0.056 | n.a. | | | 0.000/0.004 | | 0.030/0.442 | | |
| All-cause mortality | 0.845 | 0.001 | | | n.a. | | n.a. | | |
| Total expenditure | 0.083 | n.a. | | | n.a. | | n.a. | | |
| Supported accommodation expenditure | 0.013 | n.a. | | | n.a. | | n.a. | | |
| Home care expenditure | 0.000 | n.a. | | | n.a. | | n.a. | | |
| Medical expenditure | 0.218 | n.a. | | | n.a. | | n.a. | | |
| Mental health care expenditure | 0.131 | n.a. | | | n.a. | | n.a. | | |
| Working | 0.010 | 0.000 | | | 0.000/0.000 | | 0.000/0.051 | | |
| Income from work | 0.328 | 0.001 | | | 0.013/0.001 | | 0.053/0.063 | | |
| Personal Income | 0.004 | 0.018 | | | 0.000/0.000 | | 0.014/0.014 | | |

Semi-parametric test: fit-based p-value reported. n.a. (non-applicable) corresponds to outcomes that were not studied.

Table S6. Ordinary-least squares (OLS) regression results estimates for the effects of being eligible for supported housing

|  |  | **Effect of eligibility for supported housing** | | **Observations** |
| --- | --- | --- | --- | --- |
| Individual | **Calendar year after application** | coefficient | (se) |  |
|  | Supported housing admission | 0.371*** | (0.016) | 7,953 |
|  | Use of home care | -0.078*** | (0.016) | 7,953 |
|  | Use of any mental health care | 0.068*** | (0.018) | 7,852† |
|  | Total expenditure | 17,220*** | (1,188) | 7,953 |
|  | Supported housing expenditure | 15,283*** | (689) | 7,953 |
|  | Home care expenditure | -513*** | (174) | 7,953 |
|  | Medical expenditure | 31 | (263) | 7,852† |
|  | Total mental health care expenditure | 2,374** | (848) | 7,852† |
|  | Outpatient mental health care expenditure | 558 | (220) | 7,852† |
|  | Inpatient mental health care expenditure | 1,816** | (779) | 7,852† |
|  | Death for all causes | 0.002 | (0.004) | 7,953 |
|  | Working | -0.039*** | (0.014) | 7,596† |
|  | Income from work | -1,241*** | (335) | 7,596† |
|  | Personal Income | -900*** | (271) | 7,596† |
|  | **Fourth calendar year after application** |  |  |  |
|  | Death for all causes | 0.000 | (0.008) | 7,953 |
|  | Working | -0.063*** | (0.015) | 7,218† |
|  | Income from work | -2,656*** | (474) | 7,218† |
|  | Personal Income | -1,347*** | (301) | 7,218† |
| Mother | **Calendar year after application** |  |  |  |
|  | Working | 0.022 | (0.02) | 5,097 |
|  | Income from work (€) | 649 | (682) | 5,097 |
|  | Personal Income (€) | 684 | (559) | 5,097 |
|  | Use of any mental health care | 0.000 | (0.013) | 5,240 |
|  | **Fourth calendar year after application** |  |  |  |
|  | Working | 0.052** | (0.021) | 4,835 |
|  | Income from work (€) | 1,038 | (784) | 4,835 |
|  | Personal Income (€) | 262 | (664) | 4,835 |
| Father | **Calendar year after application** |  |  |  |
|  | Working | 0.013 | (0.019) | 4,061 |
|  | Income from work (€) | -434 | (1,373) | 4,061 |
|  | Personal Income (€) | 527 | (1,009) | 4,061 |
|  | Use of any mental health care | -0.025* | (0.013) | 4,169 |
|  | **Fourth calendar year after application** |  |  |  |
|  | Working | 0.009 | (0.020) | 3,765 |
|  | Income from work (€) | -740 | (1,410) | 3,765 |
|  | Personal Income (€) | 419 | (1,136) | 3,765 |

Robust standard errors in parentheses, clustered at the assessor level; *** p<0.01, ** p<0.05, * p<0.1 †The smaller number observations is due to missing data for the outcomes coming from health insurance claims and tax returns databases. All regressions include the main specification controls described in section 4.3.

Table S7. Descriptive statistics for the subpopulation with (any) parental outcome: group means for total group and by supported housing eligibility

|  | Total  (sd) | Eligible  (sd) | Non-Eligible  (sd) | Diff Non-Eligible vs Eligible | p-val  T test |
| --- | --- | --- | --- | --- | --- |
| **Individual** |  |  |  |  |  |
| Female | 0.28 | 0.28 | 0.28 | -0.00 | 0.769 |
| Age | 31.70  (11.59) | 31.81  (11.74) | 31.07  (10.65) | -0.74 | 0.089 |
| Dutch background | 0.77 | 0.77 | 0.76 | -0.01 | 0.373 |
| Western migration background† | 0.08 | 0.08 | 0.08 | -0.00 | 0.994 |
| Non-Western migration background† | 0.15 | 0.15 | 0.16 | 0.01 | 0.290 |
| Living alone | 0.41 | 0.41 | 0.41 | -0.00 | 0.865 |
| Living with partner | 0.06 | 0.06 | 0.07 | 0.01 | 0.095 |
| Living with parents | 0.21 | 0.21 | 0.18 | -0.03 | 0.033 |
| Living with single parent | 0.11 | 0.11 | 0.11 | 0.00 | 0.933 |
| Living institution | 0.17 | 0.17 | 0.16 | -0.01 | 0.410 |
| Other position in household | 0.04 | 0.04 | 0.07 | 0.03 | 0.000 |
| Prior‡ personal income (€) | 11,799  (7,415) | 11,648  (7,170) | 12,693  (8,680) | 1,044 | 0.000 |
| Prior‡ working | 0.31 | 0.30 | 0.37 | 0.06 | 0.000 |
| Prior‡ specialist mental health care | 0.67 | 0.68 | 0.59 | -0.09 | 0.000 |
| Prior‡ home care | 0.12 | 0.13 | 0.08 | -0.05 | 0.000 |
| Prior‡ medical expenditure (€) | 19,578  (37,891) | 20,849  (39,260) | 12,037  (27,273) | -8,812 | 0.000 |
| Mental health disorder diagnosis§ |  |  |  |  |  |
| Childhood | 0.22 | 0.21 | 0.27 | 0.06 | 0.000 |
| Substance | 0.18 | 0.19 | 0.07 | -0.12 | 0.000 |
| Psychotic | 0.18 | 0.19 | 0.13 | -0.06 | 0.000 |
| Mood | 0.07 | 0.07 | 0.07 | 0.01 | 0.492 |
| Personality | 0.08 | 0.08 | 0.08 | -0.01 | 0.551 |
| Other diagnosis | 0.10 | 0.10 | 0.11 | 0.01 | 0.289 |
| No treatment/diagnosis | 0.18 | 0.17 | 0.27 | 0.11 | 0.000 |
| **Application** |  |  |  |  |  |
| Application by long-term care provider | 0.78 | 0.79 | 0.73 | -0.06 | 0.000 |
| Application by social worker | 0.16 | 0.15 | 0.21 | 0.06 | 0.000 |
| Application by other¶ | 0.06 | 0.06 | 0.06 | -0.00 | 0.974 |
| Regular application | 0.96 | 0.96 | 0.96 | -0.00 | 0.549 |
| Abridged assessment procedure | 0.22 | 0.16 | 0.58 | 0.42 | 0.000 |
| Extended assessment procedure | 0.78 | 0.84 | 0.42 | -0.42 | 0.000 |
| Eligible for long-term care last month# | 0.32 | 0.33 | 0.29 | -0.04 | 0.025 |
| **Eligibility** |  |  |  |  |  |
| Supported housing | 0.86 | 1.00 | 0.00 | -1.00 | . |
| Inpatient mental health care | 0.00 | 0.00 | 0.01 | 0.01 | 0.000 |
| Elderly Nursing home | 0.00 | 0.00 | 0.00 | 0.00 | 0.010 |
| Institution Disabled | 0.00 | 0.00 | 0.02 | 0.02 | 0.000 |
| Negative decision | 0.06 | 0.00 | 0.44 | 0.44 | 0.000 |
| Any home careΔ | 0.08 | 0.01 | 0.52 | 0.51 | 0.000 |
| Nursing | 0.00 | 0.00 | 0.02 | 0.02 | 0.000 |
| Individual assistance | 0.08 | 0.01 | 0.51 | 0.51 | 0.000 |
| Group assistance | 0.01 | 0.00 | 0.08 | 0.08 | 0.000 |
| Observations | 5,727 | 4,901 | 826 | n.a. | n.a. |

sd – standard deviation †First-generation migrants are classified according to their country of birth. Second-generation migrants are classified according to their mother´s country of birth – if that is the Netherlands the father´s country of birth is considered. ‡ Prior refers to the calendar year before the year of application. §Only available for those who receive specialist mental health care in the past 365 days. ¶ Including the candidate herself. # Except for supported housing as all those eligible for supported housing in the 365 days ahead of the application were excluded. Δ The rows below refer to the different types of home care granted in our population. Individuals can be eligible for more than one type of home care simultaneously.

Table S8. Two-stage least squares (2SLS) regression estimates for the effects of being eligible for supported housing: subpopulation with (any) parental outcome.

|  | **Effect of eligibility for supported housing** | |  | **First-stage** | |  | **Observations** | **Mean dependent variable non-eligible group** |
| --- | --- | --- | --- | --- | --- | --- | --- | --- |
| **Calendar year after application** | coefficient | (se) |  | coefficient | (se) |  |  |  |
| Supported housing admission | 0.257*** | (0.057) |  | 1.073*** | (0.05) |  | 5,727 | 0.44 |
| Use of home care | -0.123** | (0.058) |  | 1.073*** | (0.05) |  | 5,727 | 0.26 |
| Use of mental health care | -0.066 | (0.060) |  | 1.067*** | (0.05) |  | 5,678† | 0.65 |
| Total expenditure (€) | 18,196*** | (5,719) |  | 1.073*** | (0.05) |  | 5,727 | 30,521 |
| Supported housing expenditure (€) | 10,491*** | (2,426) |  | 1.073*** | (0.05) |  | 5,727 | 15,719 |
| Home care expenditure (€) | 23 | (536) |  | 1.073*** | (0.05) |  | 5,727 | 1,363 |
| Medical expenditure (€) | -1,833 | (888) |  | 1.067*** | (0.05) |  | 5,678† | 2,573 |
| Total mental health care expenditure (€) | 7,919 | (4,832) |  | 1.067*** | (0.05) |  | 5,678† | 9,697 |
| Outpatient mental health care expenditure (€) | -909 | (750) |  | 1.067*** | (0.05) |  | 5,678† | 2,588 |
| Inpatient mental health care expenditure (€) | 7,009 | (4,713) |  | 1.067*** | (0.05) |  | 5,678† | 4,141 |
| All-cause mortality | -0.01 | (0.011) |  | 1.073*** | (0.05) |  | 5,727 | 0.01 |
| Working | -0.090** | (0.043) |  | 1.083*** | (0.05) |  | 5,524† | 0.26 |
| Income from work (€) | -2,016** | (946) |  | 1.083*** | (0.05) |  | 5,524† | 2,787 |
| Personal Income (€) | -1,524** | (772) |  | 1.083*** | (0.05) |  | 5,524† | 14,113 |
| **Fourth calendar year after application** |  |  |  |  |  |  |  |  |
| All-cause mortality | -0.004 | (0.024) |  | 1.073*** | (0.05) |  | 5,727 | 0.03 |
| Working | -0.079 | (0.061) |  | 1.067*** | (0.06) |  | 5,378† | 0.29 |
| Income from work (€) | -3,195** | (1,604) |  | 1.067*** | (0.06) |  | 5,378† | 5,099 |
| Personal Income (€) | -2,116** | (861) |  | 1.067*** | (0.06) |  | 5,378† | 17,040 |

Robust standard errors in parentheses, clustered at the assessor level; *** p<0.01, ** p<0.05, * p<0.1. †The smaller number observations is due to missing data for the outcomes coming from health insurance claims and tax returns databases. All regressions include the main specification controls described in section 4.3.

Table S9. Two-stage least squares (2SLS) regression estimates for the parental outcomes of being eligible for supported housing admission: subgroup living with parents prior to the application.

|  | | **Effect of eligibility for supported housing** | |  | **First-stage** | |  | **Observations** | **Mean dependent variable non-eligible group** |
| --- | --- | --- | --- | --- | --- | --- | --- | --- | --- |
| **Calendar year after application** | | coefficient | (se) |  | coefficient | (se) |  |  |  |
| **Mother** | Working | 0.039 | (0.138) |  | 1.076*** | (0.100) |  | 1,719 | 0.56 |
|  | Income from work (€) | 9,677** | (4,536) |  | 1.076*** | (0.100) |  | 1,719 | 13,435 |
|  | Personal Income (€) | 5,572 * | (3,117) |  | 1.076*** | (0.100) |  | 1,719 | 18,576 |
|  | Use of mental health care | -0.004 | (0.109) |  | 1.078*** | (0.100) |  | 1,761 | 0.14 |
| **Father** | Working | 0.275*** | (0.100) |  | 1.094*** | (0.120) |  | 1,511 | 0.71 |
|  | Income from work (€) | 7,389 | (8,620) |  | 1.094*** | (0.120) |  | 1,511 | 36,886 |
|  | Personal Income (€) | 488 | (5,897) |  | 1.094*** | (0.120) |  | 1,511 | 39,365 |
|  | Use of mental health care | 0.046 | (0.043) |  | 1.085*** | (0.076) |  | 4,169 | 0.14 |
| **Fourth calendar year after application** | |  |  |  |  |  |  |  |  |
| **Mother** | Working | 0.248 | (0.154) |  | 1.085*** | (0.100) |  | 1,684 | 0.50 |
|  | Income from work (€) | 9,146* | (5,074) |  | 1.085*** | (0.100) |  | 1,684 | 13,198 |
|  | Personal Income (€) | 3,205 | (3,579) |  | 1.085*** | (0.100) |  | 1,684 | 20,109 |
| **Father** | Working | 0.336*** | (0.119) |  | 1.084*** | (0.120) |  | 1,456 | 0.67 |
|  | Income from work (€) | 14,428 | (8,692) |  | 1.084*** | (0.120) |  | 1,456 | 36,212 |
|  | Personal Income (€) | 3,579 | (6,890) |  | 1.084*** | (0.120) |  | 1,456 | 41,030 |

Robust standard errors in parentheses, clustered at the assessor level; *** p<0.01, ** p<0.05, * p<0.1 All regressions include the main specification controls described in section 4.3.

Table S10. Two-stage least squares (2SLS)regression estimates for the effects of being eligible for supported housing adjusting for quarter of the year

|  | **Effect of eligibility for supported housing** | |  | **First-stage** | |  | **Observations** | **Mean dependent variable non-eligible group** |
| --- | --- | --- | --- | --- | --- | --- | --- | --- |
| **Calendar year after application** | coefficient | (se) |  | coefficient | (se) |  |  |  |
| Supported housing admission | 0.301*** | (0.056) |  | 0.982*** | (0.070) |  | 7,953 | 0.52 |
| Use of home care | -0.120* | (0.069) |  | 0.982*** | (0.070) |  | 7,953 | 0.26 |
| Use of mental health care | -0.049 | (0.054) |  | 0.979*** | (0.070) |  | 7,852† | 0.66 |
| Total expenditure (€) | 20,037*** | (5,780) |  | 0.982*** | (0.070) |  | 7,953 | 33,315 |
| Supported housing expenditure (€) | 11,682*** | (2,518) |  | 0.982*** | (0.070) |  | 7,953 | 17,345 |
| Home care expenditure (€) | -392 | (590) |  | 0.982*** | (0.070) |  | 7,953 | 1,513 |
| Medical expenditure (€) | -840 | (889) |  | 0.979*** | (0.070) |  | 7,852† | 3,111 |
| Total mental health care expenditure (€) | 7,162 | (5,334) |  | 0.979*** | (0.070) |  | 7,852† | 9,401 |
| Outpatient mental health care expenditure (€) | 1,107 | (782) |  | 0.979*** | (0.070) |  | 7,852† | 2,419 |
| Inpatient mental health care expenditure (€) | 6,345 | (4,795) |  | 0.979*** | (0.070) |  | 7,852† | 4,021 |
| All-cause mortality | -0.005 | (0.016) |  | 0.982*** | (0.070) |  | 7,953 | 0.02 |
| Working | -0.088** | (0.041) |  | 0.997*** | (0.060) |  | 7,596† | 0.21 |
| Income from work (€) | -2,104*** | (774) |  | 0.997*** | (0.060) |  | 7,596† | 2,317 |
| Personal Income (€) | -1,963** | (799) |  | 0.997*** | (0.060) |  | 7,596† | 14,218 |
| **Fourth calendar year after application** |  |  |  |  |  |  |  |  |
| All-cause mortality | 0.048 | (0.033) |  | 0.982*** | (0.070) |  | 7,953 | 0.06 |
| Working | -0.105* | (0.054) |  | 0.999*** | (0.070) |  | 7,218† | 0.24 |
| Income from work (€) | -3,253** | (1,479) |  | 0.999*** | (0.070) |  | 7,218† | 4,164 |
| Personal Income (€) | -2,653*** | (767) |  | 0.999*** | (0.070) |  | 7,218† | 16,718 |

Robust standard errors in parentheses, clustered at the assessor level; *** p<0.01, ** p<0.05, * p<0.1. †The smaller number observations is due to missing data for the outcomes coming from health insurance claims and tax returns databases. All regressions include the main specification controls described in section 4.3, except for half-year dummies, that were replaced by quarter of the year dummies.

Table S11. Unbiased jackknife instrumental variables estimates using assessor-type of assessment fixed effects: effects of being eligible for supported housing on individual outcomes

|  | **Effect of eligibility for supported housing** | |  | **Observations** |  | | **F-stage F-statistics** | | | **Mean dependent variable non-eligible group** |
| --- | --- | --- | --- | --- | --- | --- | --- | --- | --- | --- |
| **Calendar year after application** | coefficient | (se) |  |  | |  | |  |  | |
| Supported housing admission | 0.341*** | (0.073) |  | 7,953 | |  | | 4.178 | 0.52 | |
| Use of home care | -0.088 | (0.085) |  | 7,953 | |  | | 4.178 | 0.26 | |
| Use of mental health care | 0.006 | (0.081) |  | 7,852† | |  | | 4 146 | 0.66 | |
| Total expenditure (€) | 25,601*** | (6,032) |  | 7,953 | |  | | 4.178 | 33,315 | |
| Supported housing expenditure (€) | 12,003*** | (2,693) |  | 7,953 | |  | | 4.178 | 17,345 | |
| Home care expenditure (€) | -963 | (814) |  | 7,953 | |  | | 4.178 | 1,513 | |
| Medical expenditure (€) | -188 | (1,053) |  | 7,852† | |  | | 4.146 | 3,111 | |
| Total mental health care expenditure (€) | 14,082** | (5,575) |  | 7,852† | |  | | 4.146 | 9,401 | |
| Outpatient mental health care expenditure (€) | 1,467 | (1,261) |  | 7,852† | |  | | 4.146 | 3,111 | |
| Inpatient mental health care expenditure (€) | 1,261 | (5,248) |  | 7,852† | |  | | 4.146 | 9,401 | |
| All-cause mortality | -0.018 | (0.022) |  | 7,953 | |  | | 4.178 | 0.02 | |
| Working | -0.187*** | (0.063) |  | 7,596† | |  | | 4.144 | 0.21 | |
| Income from work (€) | -2,515** | (1,176) |  | 7,596† | |  | | 4.144 | 2,317 | |
| Personal Income (€) | -2,495** | (1,039) |  | 7,596† | |  | | 4.144 | 14,218 | |
| **Fourth calendar year after application** |  |  |  |  | |  | |  |  | |
| All-cause mortality | 0.061 | (0.038) |  | 7,953 | |  | | 4.178 | 0.06 | |
| Working | -0.217*** | (0.070) |  | 7,218† | |  | | 4.112 | 0.24 | |
| Income from work (€) | -5,639*** | (1,553) |  | 7,218† | |  | | 4.112 | 4,164 | |
| Personal Income (€) | -4,671*** | (1,255) |  | 7,218† | |  | | 4.112 | 16,718 | |

Robust standard errors in parentheses, clustered at the assessor level; *** p<0.01, ** p<0.05, * p<0.1. †The smaller number observations is due to missing data for the outcomes coming from health insurance claims and tax returns databases. All regressions include the main specification controls described in section 4.3. The smaller number observations is due to missing data for the outcomes coming from health insurance claims and tax returns databases.

Table S12. Unbiased jackknife instrumental variables estimates using assessor-type of assessment fixed effects: effects of being eligible for supported housing admission on parental outcomes

|  | | **Effect of eligibility for supported housing** | |  | **Observations** |  | | **F-stage F-statistics** | | | **Mean dependent variable non-eligible group** |
| --- | --- | --- | --- | --- | --- | --- | --- | --- | --- | --- | --- |
| **Calendar year after application** | | coefficient | (se) |  |  | |  | |  |  | |
| **Mother** | Working | -0.018 | (0.075) |  | 5,097 | |  | | 3.891 | 0.40 | |
|  | Income from work (€) | 2,505 | (2,154) |  | 5,097 | |  | | 3.891 | 9,749 | |
|  | Personal Income (€) | 734 | (1,876) |  | 5,097 | |  | | 3.891 | 17,161 | |
|  | Use of mental health care | 0.019 | (0.056) |  | 5,077 | |  | | 3.858 | 0.12 | |
| **Father** | Working | 0.216** | (0.090) |  | 4,061 | |  | | 3.471 | 0.54 | |
|  | Income from work (€) | 7,311 | (5,971) |  | 4,061 | |  | | 3.471 | 25,877 | |
|  | Personal Income (€) | 6,258 | (4,827) |  | 4,061 | |  | | 3.471 | 34,651 | |
|  | Use of mental health care | 0.046 | (0.044) |  | 4,026 | |  | | 3.420 | 0.08 | |
| **Fourth calendar year after application** | |  |  |  |  | |  | |  |  | |
| **Mother** | Working | -0.006 | (0.075) |  | 4,835 | |  | | 3.713 | 0.36 | |
|  | Income from work (€) | -1,247 | (2,704) |  | 4,835 | |  | | 3.713 | 9,779 | |
|  | Personal Income (€) | -1,772 | (2,425) |  | 4,835 | |  | | 3.713 | 18,629 | |
| **Father** | Working | 0.355*** | (0.094) |  | 3,765 | |  | | 3.186 | 0.51 | |
|  | Income from work (€) | 9,047 | (6,289) |  | 3,765 | |  | | 3.186 | 25,799 | |
|  | Personal Income (€) | 4,576 | (4,673) |  | 3,765 | |  | | 3.186 | 36,505 | |

Robust standard errors in parentheses, clustered at the assessor level; *** p<0.01, ** p<0.05, * p<0.1. All regressions include the main specification controls described in section 4.3.

Table S13. Descriptive statistics by age: group means for study population and by age groups

|  | Total | 18-21 years-old | 22-30  years-old | 31-50  years-old | 51-70  years-old |
| --- | --- | --- | --- | --- | --- |
| **Individual** |  |  |  |  |  |
| Female | 0.29 | 0.37 | 0.27 | 0.24 | 0.33 |
| Age | 37.73 | 19.61 | 24.96 | 39.68 | 59.27 |
| Dutch background | 0.73 | 0.81 | 0.74 | 0.67 | 0.74 |
| Western migration background† | 0.10 | 0.08 | 0.08 | 0.10 | 0.12 |
| Non-Western migration background† | 0.18 | 0.11 | 0.18 | 0.23 | 0.14 |
| Living alone | 0.46 | 0.25 | 0.36 | 0.53 | 0.58 |
| Living with partner | 0.07 | 0.03 | 0.05 | 0.08 | 0.10 |
| Living with parents | 0.15 | 0.37 | 0.28 | 0.06 |  |
| Living with single parent | 0.08 | 0.19 | 0.13 | 0.04 | 0.01 |
| Living institution | 0.20 | 0.12 | 0.11 | 0.24 | 0.27 |
| Other position in household | 0.05 | 0.04 | 0.06 | 0.04 | 0.04 |
| Prior ‡ personal income (€) | 12,414 | 6,191 | 12,382 | 13,571 | 15,553 |
| Prior ‡ working | 0.25 | 0.51 | 0.36 | 0.18 | 0.08 |
| Prior ‡ income from work | 2,034 | 1,701 | 2,769 | 2,298 | 1,302 |
| Prior ‡ specialist mental health care | 0.67 | 0.61 | 0.65 | 0.71 | 0.65 |
| Prior ‡ home care | 0.15 | 0.05 | 0.09 | 0.15 | 0.29 |
| Prior ‡ health care expenditure (€) | 21,058 | 12,687 | 18,913 | 22,520 | 27,135 |
| Mental health disorder diagnosis § |  |  |  |  |  |
| Childhood | 0.24 | 0.06 | 0.16 | 0.34 | 0.27 |
| Substance | 0.19 | 0.05 | 0.17 | 0.25 | 0.24 |
| Psychotic | 0.13 | 0.36 | 0.20 | 0.05 | 0.02 |
| Mood | 0.08 | 0.06 | 0.07 | 0.06 | 0.15 |
| Personality | 0.08 | 0.06 | 0.09 | 0.09 | 0.07 |
| Other diagnosis | 0.10 | 0.15 | 0.10 | 0.08 | 0.08 |
| No treatment/diagnosis | 0.18 | 0.26 | 0.20 | 0.13 | 0.16 |
| **Application** |  |  |  |  |  |
| Application by long-term care provider | 0.79 | 0.72 | 0.74 | 0.84 | 0.83 |
| Application by social worker | 0.14 | 0.23 | 0.20 | 0.09 | 0.08 |
| Application by other ¶ | 0.07 | 0.05 | 0.07 | 0.07 | 0.09 |
| Regular application | 0.96 | 0.94 | 0.95 | 0.98 | 0.98 |
| Abridged assessment procedure | 0.24 | 0.20 | 0.20 | 0.24 | 0.32 |
| Extended assessment procedure | 0.76 | 0.80 | 0.80 | 0.76 | 0.68 |
| Eligible for long-term care last month # | 0.34 | 0.24 | 0.35 | 0.33 | 0.42 |
| **Eligibility** |  |  |  |  |  |
| Supported housing | 0.86 | 0.86 | 0.84 | 0.85 | 0.88 |
| Inpatient mental health care | 0.00 | 0.00 | 0.00 | 0.00 | 0.00 |
| Elderly Nursing home | 0.00 | 0.00 | 0.00 | 0.00 | 0.02 |
| Institution Disabled | 0.00 | 0.00 | 0.00 | 0.00 | 0.00 |
| Negative decision | 0.06 | 0.06 | 0.08 | 0.07 | 0.03 |
| Any home care Δ | 0.08 | 0.08 | 0.09 | 0.09 | 0.07 |
| Nursing | 0.01 | 0.00 | 0.00 | 0.00 | 0.01 |
| Individual assistance | 0.08 | 0.08 | 0.09 | 0.08 | 0.06 |
| Group assistance | 0.02 | 0.01 | 0.01 | 0.02 | 0.02 |
| Observations (%) | 7,953 | 19.04% | 19.86% | 36.65% | 24.4% |

†First-generation migrants are classified according to their country of birth. Second-generation migrants are classified according to their mother´s country of birth – if that is the Netherlands the father´s country of birth is considered. ‡ Prior refers to the calendar year before the year of application. §Only available for those who receive specialist mental health care in the past 365 days. ¶ Including the candidate herself. # Except for supported housing as all those eligible for supported housing in the 365 days ahead of the application were excluded. Δ The rows below refer to the different types of home care granted in our population. Individuals can be eligible for more than one type of home care simultaneously.

Table S14. Two-stage least squares (2SLS) regression estimates for individual outcomes with alternative treatment definition: supported housing + institutional care

|  | **Effect of eligibility for supported housing** | |  | **First-stage** | |  | | **Observations** |
| --- | --- | --- | --- | --- | --- | --- | --- | --- |
| **Calendar year after application** | coefficient | (se) |  | coefficient | (se) |  | |  |
| Supported housing admission | 0.354*** | (0.056) |  | 1.062*** | (0.043) | |  | 7,953 |
| Use of home care | -0.138** | (0.064) |  | 1.062*** | (0.043) | |  | 7,953 |
| Use of mental health care | -0.04 | (0.049) |  | 1.060*** | (0.043) | |  | 7,852† |
| Total expenditure (€) | 21,690*** | (4,708) |  | 1.062*** | (0.043) | |  | 7,953 |
| Supported housing expenditure (€) | 13,649*** | (2,446) |  | 1.062*** | (0.043) | |  | 7,953 |
| Home care expenditure (€) | -178 | (512) |  | 1.062*** | (0.043) | |  | 7,953 |
| Medical expenditure (€) | -806 | (855) |  | 1.060*** | (0.043) | |  | 7,852† |
| Total mental health care expenditure (€) | 7,244* | (3,903) |  | 1.060*** | (0.043) | |  | 7,852† |
| Outpatient mental health care expenditure (€) | 980 | (617) |  | 1.060*** | (0.043) | |  | 7,852† |
| Inpatient mental health care expenditure (€) | 6,263 | (3,894) |  | 1.060*** | (0.043) | |  | 7,852† |
| All-cause mortality | -0.016 | (0.013) |  | 1.062*** | (0.043) | |  | 7,953 |
| Working | -0.085* | (0.037) |  | 1.073*** | (0.043) | |  | 7,596† |
| Income from work (€) | -2,425*** | (695) |  | 1.073*** | (0.043) | |  | 7,596† |
| Personal Income (€) | -1,490** | (713) |  | 1.073*** | (0.043) | |  | 7,596† |
| **Fourth calendar year after application** |  |  |  |  |  | |  |  |
| All-cause mortality | 0.034 | (0.029) |  | 1.062*** | (0.043) | |  | 7,953 |
| Working | -0.087 | (0.052) |  | 1.075*** | (0.040) | |  | 7,218† |
| Income from work (€) | -3,763* | (1,435) |  | 1.075*** | (0.040) | |  | 7,218† |
| Personal Income (€) | -2,276*** | (718) |  | 1.075*** | (0.040) | |  | 7,218† |

Robust standard errors in parentheses, clustered at the assessor level; *** p<0.01, ** p<0.05, * p<0.1. †The smaller number observations is due to missing data for the outcomes coming from health insurance claims and tax returns databases. All regressions include the main specification controls described in section 4.3. Estimates above are results of 2SLS when the instrument is defined as eligibility to supported housing or institutional care (inpatient mental health care, nursing homes or residences for the disabled). Results are similar to main estimates, reinforcing the idea that the small share of individuals from the control group that is granted eligibility to institutional care has little influence the LATEs of our main specification.


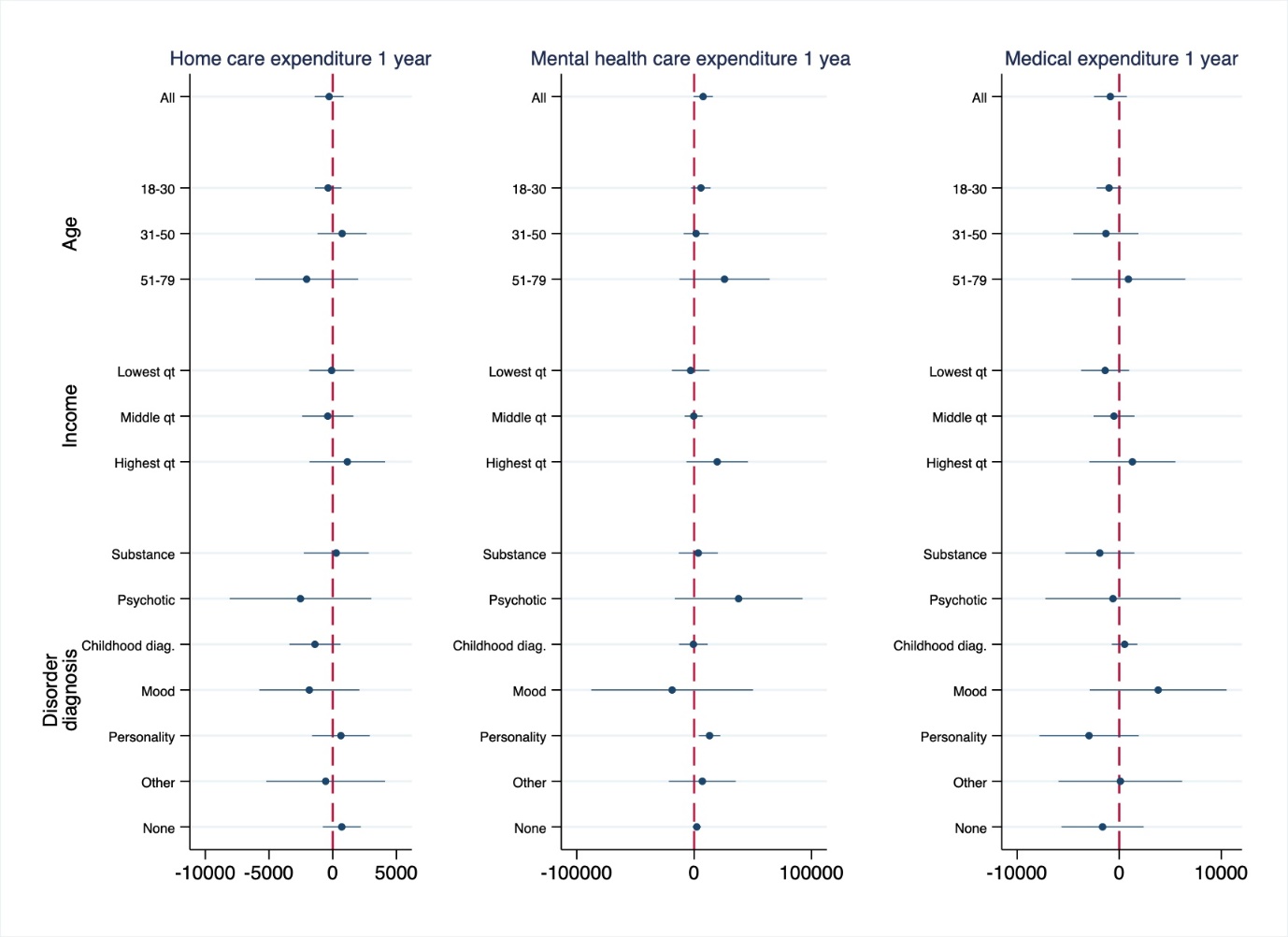

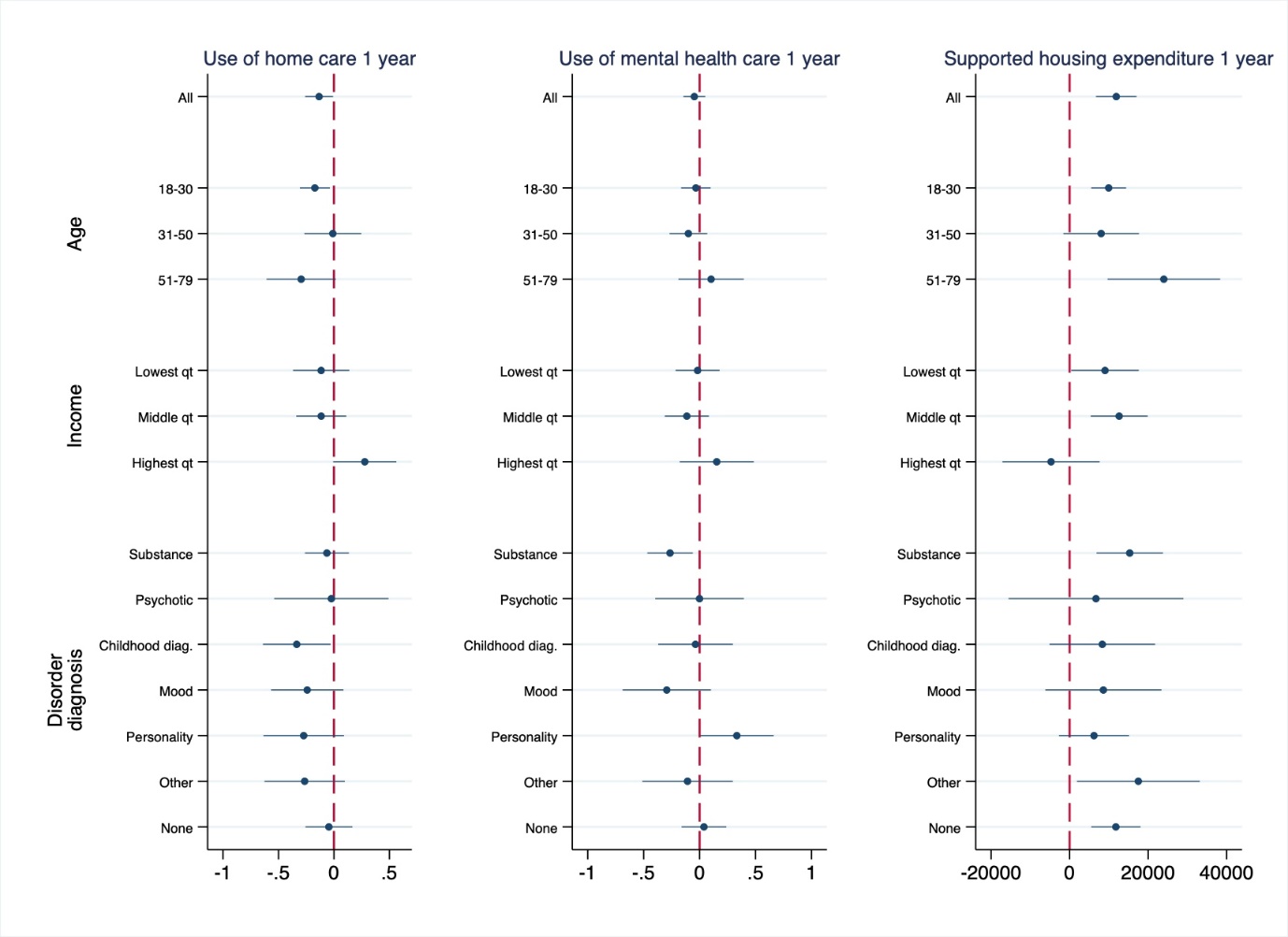


Figure S1. Subgroup analysis for additional outcomes


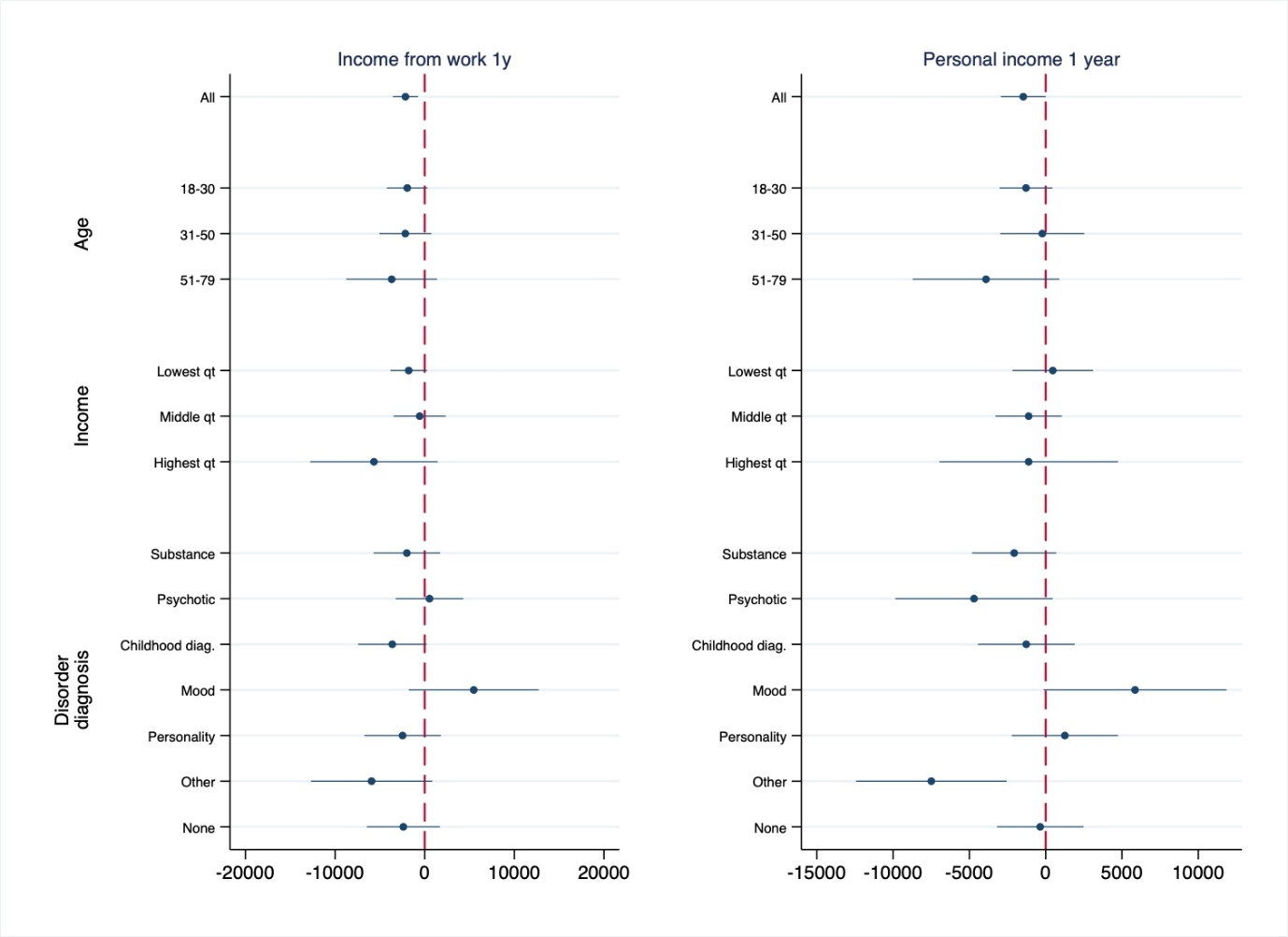


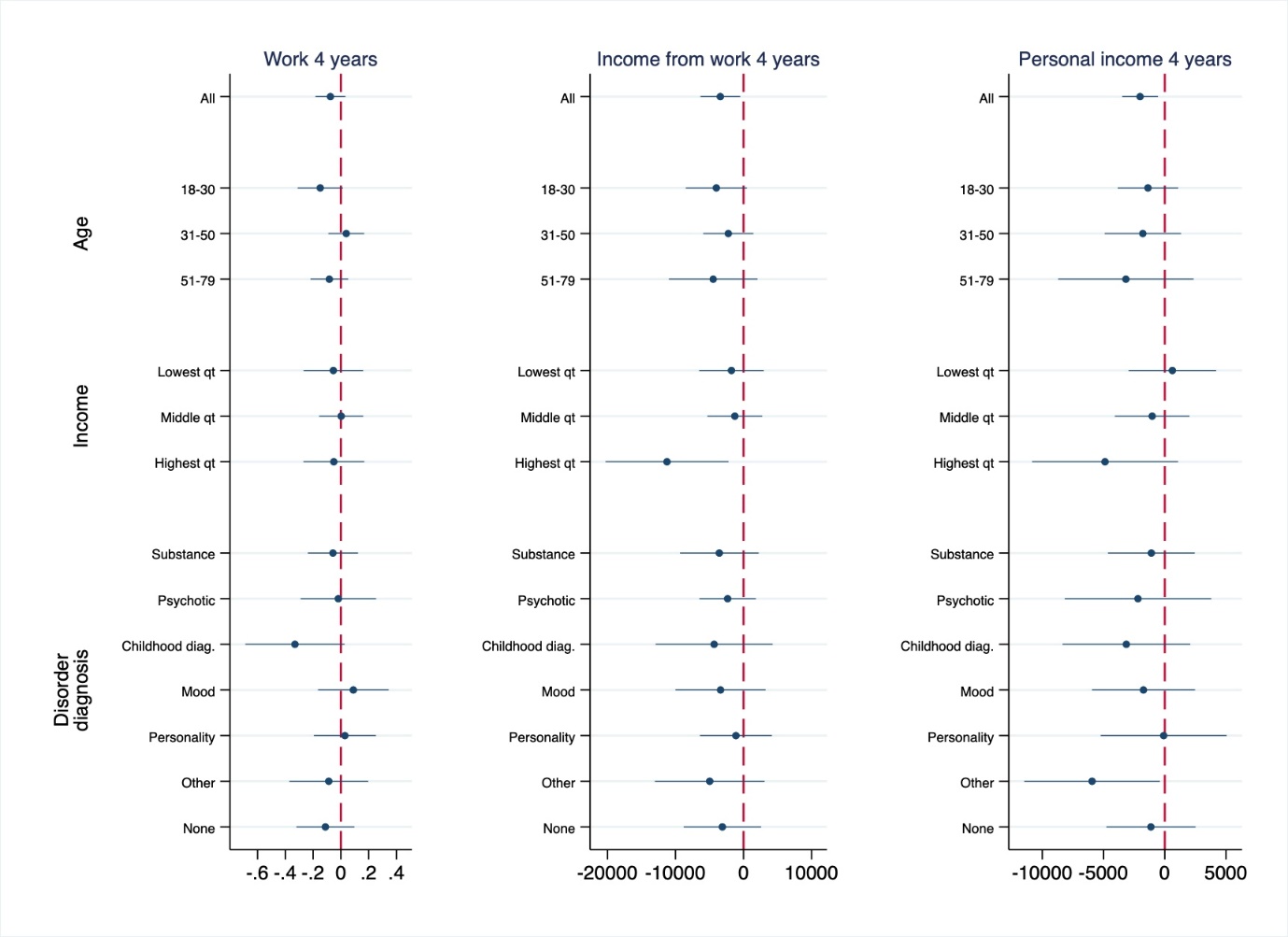


Figure S1. Subgroup analysis for additional outcomes (continuation).


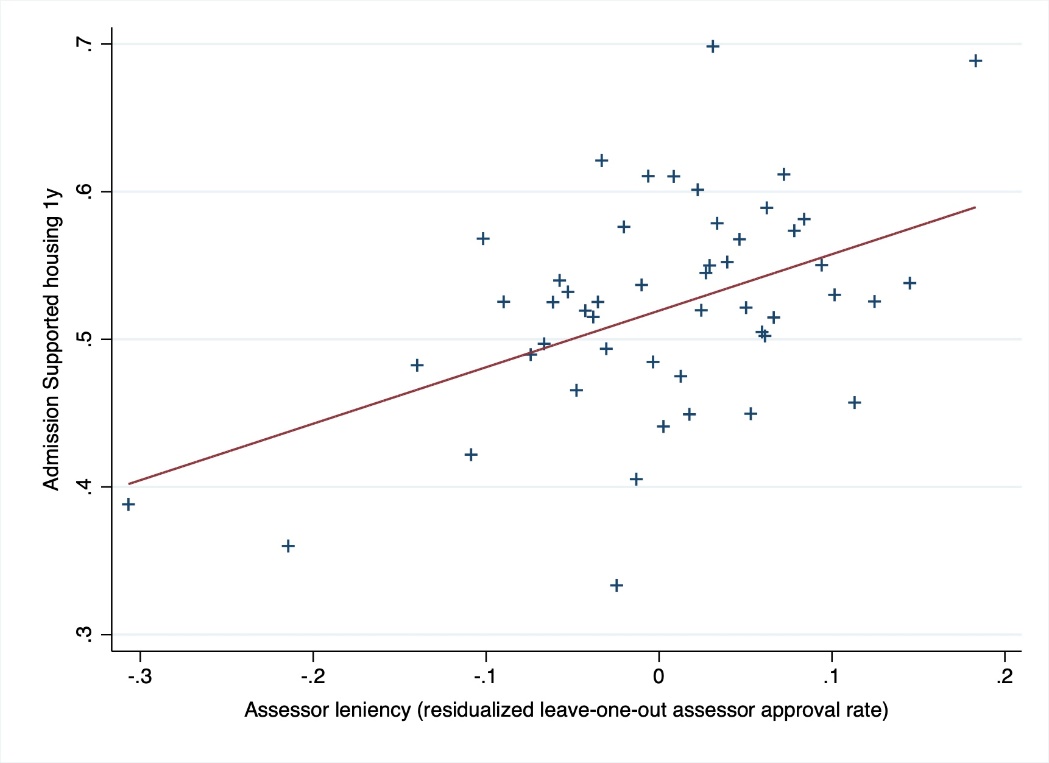


Figure S2. Residualized assessor leniency and admission to supported housing.

Note: Each dot corresponds to the likelihood of being admitted to institutional care, averaged for 1/50 parts of the population ranked by assessors leniency.


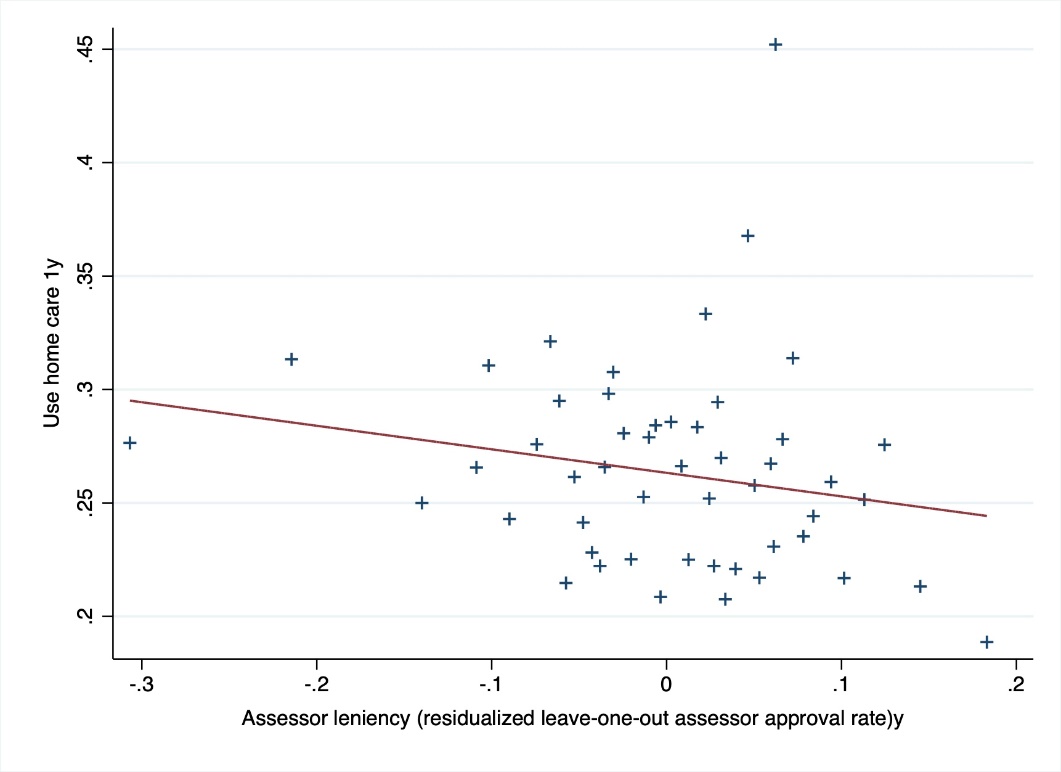


Figure S3. Residualized assessor leniency and use of home care.

Note: Each dot corresponds to the likelihood of being admitted to institutional care, averaged for 1/50 parts of the population ranked by assessors leniency.


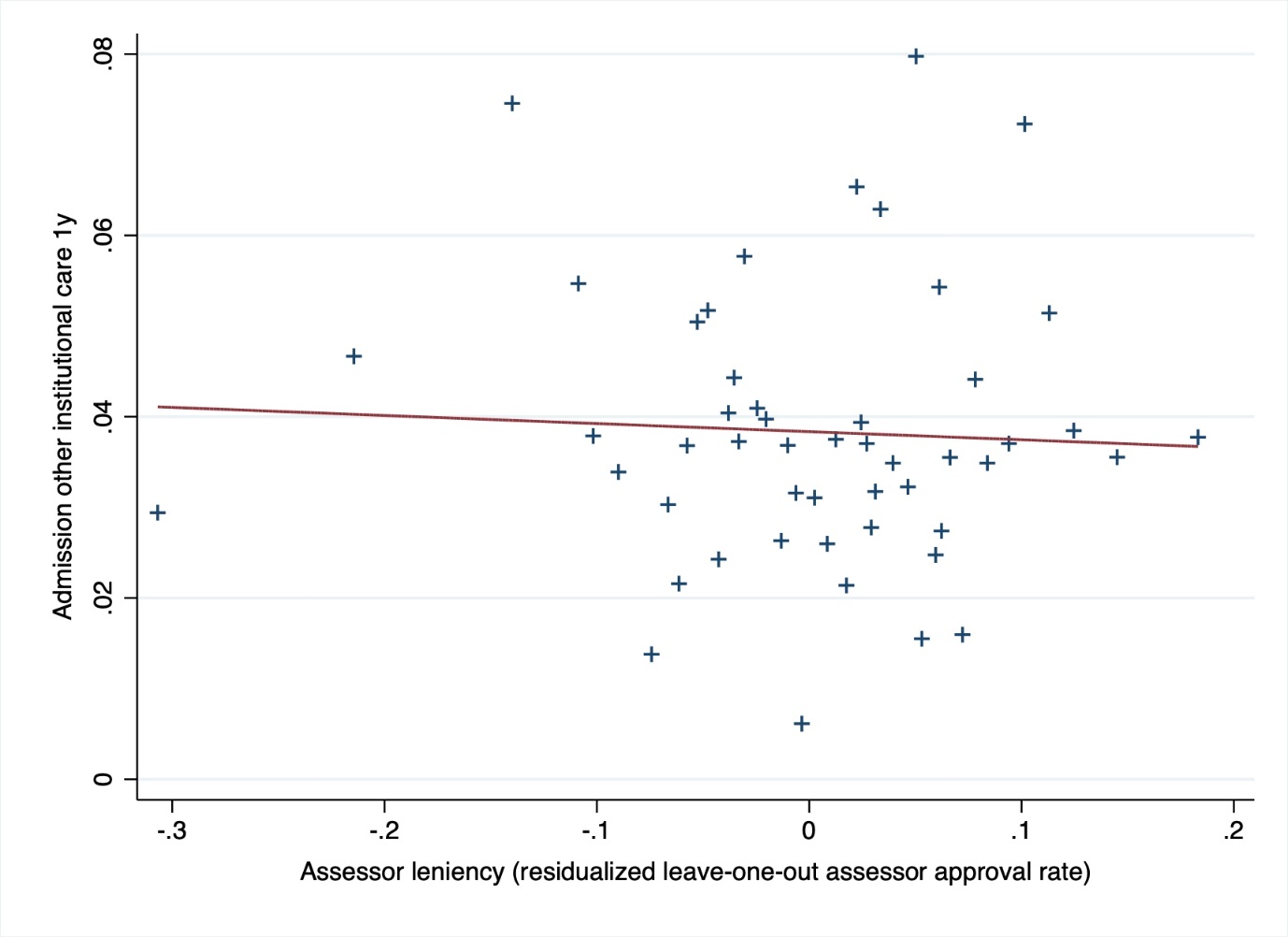


Figure S4. Residualized assessor leniency and admission to institutional care.

Note: Each dot corresponds to the likelihood of being admitted to institutional care, averaged for 1/50 parts of the population ranked by assessors leniency.
